# Comparative analysis of *Salmonella* susceptibility and tolerance to the biocide chlorhexidine identifies a complex cellular defense network

**DOI:** 10.3389/fmicb.2014.00373

**Published:** 2014-08-01

**Authors:** Orla Condell, Karen A. Power, Kristian Händler, Sarah Finn, Aine Sheridan, Kjell Sergeant, Jenny Renaut, Catherine M. Burgess, Jay C. D. Hinton, Jarlath E. Nally, Séamus Fanning

**Affiliations:** ^1^UCD Centre for Food Safety, School of Public Health, Physiotherapy and Population Science, University College DublinBelfield, Dublin, Ireland; ^2^European Program for Public Health Microbiology Training, European Centre for Disease Prevention and ControlStockholm, Sweden; ^3^Department of Microbiology, School of Genetics and Microbiology, Moyne Institute of Preventive Medicine, Trinity College DublinDublin, Ireland; ^4^Food Safety Department, Teagasc Food Research CentreAshtown, Dublin, Ireland; ^5^Department of Environment and Agrobiotechnologies (EVA), Centre de Recherche Public-Gabriel LippmannBelvaux, Luxembourg; ^6^Institute of Integrative Biology, University of LiverpoolLiverpool, UK; ^7^School of Veterinary Medicine, University College DublinBelfield, Dublin, Ireland; ^8^Institute for Global Food Security, Queen's University BelfastBelfast, Northern Ireland

**Keywords:** *Salmonella*, biocide tolerance, chlorhexidine, proteomics, transcriptomics, whole genome sequencing, SNP typing

## Abstract

Chlorhexidine is one of the most widely used biocides in health and agricultural settings as well as in the modern food industry. It is a cationic biocide of the biguanide class. Details of its mechanism of action are largely unknown. The frequent use of chlorhexidine has been questioned recently, amidst concerns that an overuse of this compound may select for bacteria displaying an altered susceptibility to antimicrobials, including clinically important anti-bacterial agents. We generated a *Salmonella enterica* serovar Typhimurium isolate (ST24^CHX^) that exhibited a high-level tolerant phenotype to chlorhexidine, following several rounds of *in vitro* selection, using sub-lethal concentrations of the biocide. This mutant showed altered suceptibility to a panel of clinically important antimicrobial compounds. Here we describe a genomic, transcriptomic, proteomic, and phenotypic analysis of the chlorhexidine tolerant *S.* Typhimurium compared with its isogenic sensitive progenitor. Results from this study describe a chlorhexidine defense network that functions in both the reference chlorhexidine sensitive isolate and the tolerant mutant. The defense network involved multiple cell targets including those associated with the synthesis and modification of the cell wall, the SOS response, virulence, and a shift in cellular metabolism toward anoxic pathways, some of which were regulated by CreB and Fur. In addition, results indicated that chlorhexidine tolerance was associated with more extensive modifications of the same cellular processes involved in this proposed network, as well as a divergent defense response involving the up-regulation of additional targets such as the flagellar apparatus and an altered cellular phosphate metabolism. These data show that sub-lethal concentrations of chlorhexidine induce distinct changes in exposed *Salmonella*, and our findings provide insights into the mechanisms of action and tolerance to this biocidal agent.

## Introduction

Chlorhexidine (1,6 *bis*(4′-chlorophenylbiguanide)hexane) is a cationic biocide of the biguanide class. It has a broad spectrum of action and is effective against Gram-negative and Gram-positive bacteria as well as yeasts, protozoa and some lipophilic viruses (Jones, 2000). This biocide is used as a disinfectant, an antiseptic and a preservative. The application of chlorhexidine in both medical and agricultural settings, along with its use as an active component in domestic cleaning agents, is increasing (Nde et al., 2009).

Chlorhexidine has been in use for over 50 years (Block and Furman, 2002; Gilbert and Moore, 2005). Despite its broad use, precise details of its mode of action are lacking (Jones, 2000; Galice et al., 2006). Nonetheless the general mechanism of action for chlorhexidine, like other cationic compounds, involves the bacterial cell membrane (Gilbert and Moore, 2005). In Gram-negative bacteria, chlorhexidine can bind to divalent cations such as Ca^2+^ and Mg^2+^ normally associated with lipopolysaccharide, and to negatively charged proteins and acidic phospholipids (Jones, 2000). Chlorhexidine is composed of a six carbon chain, a structure that is smaller compared to other cationic compounds. In addition it contains an inflexible hydrophobic region. Its size and molecular inflexibility are thought to limit its ability to fold sufficiently to allow its insertion into the membrane (Gilbert and Moore, 2005). It has been hypothesized that chlorhexidine can form a molecular bridge between adjacent phospholipid head groups thereby displacing any divalent cations. Consequently, this binding event can alter membrane fluidity and the activity of membrane-bound enzymes and osmoregulation mechanisms (Gilbert and Moore, 2005). Chlorhexidine treatment has been associated with the loss of membrane integrity, causing widespread membrane damage, and at high concentrations, the formation of large phosphate containing moieties, which may lead to the precipitation of cytoplasmic content, including the coagulation and precipitation of nucleic acids, proteins, and the leakage of cellular materials (Jones, 2000; Hope and Wilson, 2004; Cheung et al., 2012). It is thought that chlorhexidine may also inhibit oxygen utilization (Barrett-Bee et al., 1994) and energy production in bacteria (Nde et al., 2009). Other effects caused by this biocide include DNA damage (Yeung et al., 2007; Nde et al., 2009) and the inhibition of protein synthesis (Nde et al., 2009).

Like other biocides, the wide-spread use of chlorhexidine has been questioned recently, due to the possibility that the overuse of such agents may select for mutants displaying a reduced susceptibility to it and other related and non-related antimicrobials, including clinically important agents (Gilbert and Moore, 2005). Bacteria demonstrating a reduced susceptibility to chlorhexidine have been documented (Thomas and Stickler, 1979; Kropinski et al., 1982). Chlorhexidine tolerance has been selected *in vitro* in a number of different bacterial species including *Salmonella* (Braoudaki and Hilton, 2004; Condell et al., 2012a), *Escherichia coli* (Braoudaki and Hilton, 2004) and *Staphylococcus aureus* (Kaatz et al., 2005; Huet et al., 2008). Chlorhexidine tolerance has been reported to result from modification/(s) in cell wall structure and/or composition (Russell and Furr, 1986; Russell and Path, 1986; Tattawasart et al., 2000; Denyer and Maillard, 2002), and has also been associated with an over-expression of efflux pumps (Levy, 2000; Poole, 2004; Huet et al., 2008). In some instances the tolerant phenotype that develops following chlorhexidine exposure is associated with a reduced susceptibility to clinically important antimicrobial agents (Braoudaki and Hilton, 2004; Condell et al., 2012a).

*Salmonella* is an important food-borne pathogen and the causative agent of typhoid fever and gastroenteritis in humans (Coburn et al., 2007). It can survive in non-host environments, including a range of food matrices (Gast and Holt, 1998; Shi et al., 2007) and the food processing environment itself (Nesse et al., 2003; Vestby et al., 2009). The elimination of *Salmonella* through effective control measures, including adequate hygienic cleaning and sanitation, is essential to reduce its transmission and impact on public health.

Following several rounds of *in vitro* selection using sub-lethal concentrations of chlorhexidine, a *S.* Typhimurium strain was recovered, which had developed a high-level chlorhexidine tolerance phenotype (ST24^CHX^); a >50-fold increase in the minimum inhibitory concentration (MIC) (Condell et al., 2012a). In this study, we compared the susceptible *Salmonella* Typhimurium (ST24^WT^) with ST24^CHX^ its isogenic chlorhexidine tolerant counterpart using a variety of molecular techniques, facilitating a study of the classical biochemical pathway. The alterations identified based on these approaches were subsequently validated. Using these techniques, several cellular signals involved in the bacterial response to chlorhexidine exposure were identified. Our findings provide new insights into the mechanisms of action of this biocidal agent.

## Materials and methods

### Bacterial culture, *in vitro* mutant selection, DNA, RNA, and protein preparation

All *Salmonella* isolates were stored on beads in cryopreservation fluid at −80°C (Technical Service Consultants Ltd, Lancashire, England). Isolates were streaked onto Mueller-Hinton agar (Oxoid, Cambridge, UK). A single colony was picked and subsequently used to inoculate Mueller-Hinton (MH) broth (Oxoid, Cambridge, UK), then incubated for 16–18 h at 37°C shaking at 250 rpm. The culture was used to inoculate fresh aliquots of MH broth at a dilution of 1:100. Cultures were harvested at the mid-logarithmic growth phase (OD_610 nm_ = 0.6) for the assays described below.

Based on earlier work, a *Salmonella enterica* serovar Typhimurium isolate, denoted as ST24^WT^, was chosen for further study (Condell et al., 2012a); this isolate demonstrated a reduced susceptibility to three food-grade biocide formulations that were significantly above the mean MIC for a collection of 189 *Salmonella* isolates. When tested against chlorhexidine, ST24^WT^ displayed an MIC of 1.96 μg/ml. Following several rounds of *in vitro* selection using sub-lethal concentrations of chlorhexidine (Condell et al., 2012a), a high-level tolerance phenotype was developed with an increase in the MIC to >50 μg/ml being noted. The isogenic, chlorhexidine tolerant, mutant was designated ST24^CHX^ (Condell et al., 2012a). Chlorhexidine is commercially available at concentrations from 0.5 through 4% and in a variety of formulations for healthcare applications (Milstone et al., 2008).

For purification of DNA, a 5 ml aliquot of the 16–18 h culture in MH broth (as outlined above) was removed. For purification of temporal RNA and protein 50 ml of the mid-logarithmic growth phase (outlined above) was divided in two. One-half was untreated whilst the second-half was exposed to chlorhexidine. Both ST24^WT^ and ST24^CHX^ were exposed to 0.5 × MIC of the reference strain ST24^WT^, equivalent to 1 μg/ml chlorhexidine, and incubated for 30 min at 37°C. Following the incubation period 25 ml volumes from each sample were removed for proteomic analysis (see below). Additionally 2 ml from each culture was removed for RNA purification (see below). All samples were prepared in triplicate on independent occasions.

### Genomic DNA isolation and single nucleotide polymorphism (SNP) analysis

Genomic DNA was isolated, for both ST24^WT^ and ST24^CHX^, using the DNeasy blood and tissue kit (Qiagen, Inc., Valencia, California), following the manufacturers' instructions. The isolated DNA was submitted for commercial whole genome sequencing. Illumina 100 bp paired end sequences for each sample were generated from the HiSeq platform (Source BioScience, Nottingham, UK), with a mean of 3405 Mbases. Only those reads ≥Q30 were used for downstream analysis. MAQ (Li et al., 2008) version 0.4.7 was used to map reads to, and to generate SNPs from, the reference genome *Salmonella enterica* subsp. *enterica* serovar Typhimurium *str*. SL1344 (FQ_312003). In-house perl scripts were used to annotate the resulting SNP and indel files. Locations of SNPs within genes were confirmed using the *Salmonella* Typhimurium SL1344 transcriptional map provided by Kröger et al. (2012). A summary of the detected SNPs is provided in Table 1.

**Table 1:** **Single nucleotide polymorphisms (SNPs) that differentiate the reference, chlorhexidine susceptible, *Salmonella* Typhimurium ST24^WT^ and the isogenic, chlorhexidine tolerant, mutant ST24^CHX^ detected (A) within coding genes and (B) in intergenic regions**.

**(A)**											
**Position**	Nucleotide^*^			**Gene name**	**Reference ID**	**Gene locations**	**Strand**	ST24^**WT**^ aa^*^ (codon)	ST24^**CHX**^ aa^*^ (codon)	**Gene function**	**Functional category**
	**SL1344**	**ST24^**WT**^**	**ST24^**CHX**^**								
**NON-SYNONYMOUS**											
319066	G	G	C	*sciK1*	SL1344_0274	318655..319140	+	V(GTG)	Q(CAG)	SPI-6 associated, Hcp like protein, component of type VI secretory system	Virulence
319067	T	T	A	*sciK1*	SL1344_0274	318655..319140	+	V(GTG)			
3504469	T	T	G	*lptC*	SL1344_3289	3503949..3504524	+	L(CTG)	R(CGG)	Lipopolysaccharide export system protein, forms ABC transporter with LptBFG	Transport and permeability
638182	C	C	T	*ramR*	SL1344_0568	637634..638215	−	A(GCA)	T(ACA)	TetR family transcriptional regulator, repressor of ramA	Drug transport/ metabolism
3781244	G	G	A	*pitA*	SL1344_3554	3779941..3781314	+	W(TGG)	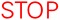 (TAG)	Low-affinity inorganic phosphate transporter, PiT family	Inorganic ion transport and metabolism
2502787	G	A	G	*yfdC*	SL1344_2362	2502523..2503464	+	G(GGC)	S(AGC)	Putative formate/nitrate transporter	Anaerobic metabolism, transport and permeability
3542329	C	T	C	*oadA2*	SL1344_3324	3541466..3543241	−	G(GGC)	S(AGC)	Oxaloacetate decarboxylase subunit alpha, involved in pyruvate metabolism, conversion of oxaloacetate to pyruvate and carbon dioxide	Amino acid metabolism, energy production
4182738	A	A	T	−	SL1344_4465	4812650..4815415	+	Y(TAT)	F(TTT)	NtrC family of transcriptional regulator, involved in activation of nitrogen metabolism related promoters and sigma 54	Regulation
4812741	T	C	T					L(CTG)	P(CCG)		
2043305	A	C	A	−	SL1344_1929	2041795..2042394		V(GTT)	A(GCT)	Bacteriophage SLP203, putative bacteriophage tail protein	Bacteriophage
2043305	A	C	A	−	SL1344_1930	2042364..2043959	−	S(TCT)	A(GCT)	Bacteriophage SLP203, putative bacteriophage tail fiber protein	Bacteriophage
2042368	A	A	G				−	V(GTA)	G(GGA)		
2045590	T	T	C	−	SL1344_1934	2045576..2045989	−	K(AAA)	Q(CAA)	Bacteriophage SLP203, bacteriophage protein	Bacteriophage
2066438	C	A	C	−	SL1344_1960	2065512..2066507	−	A(GCG)	S(TCG)	Bacteriophage SLP203, bacteriophage protein	Bacteriophage
2766226	A	A	G	*gpP*	SL1344_2590	2766099..2766791	−	V(GTT)	A(GCT)	Bacteriophage SLP272, replication protein P	Bacteriophage
**DEGENERATIVE**											
334377	C	S	C	−	SL1344_0286	332521..336123	+	G(GGC)		SPI 6 associated. Rhs family protein- membrane protein of unknown function	Virulence
3032926	G	R	G	*avrA*	SL1344_2845	3032477..3033382	−	L(CTG)		SPI 1 associated. Type III secretion system effector protein- regulator	Virulence
2509924	C	S	C	*pgtP*	SL1344_2367	2509846..2511237	+	R(CGT)		Phosphoglycerate transporter protein, induced by pgtBC	Inorganic ion transport and metabolism
4404698	T	K	T	*nudC*	SL1344_4105	4404662..4405435	+	W(TGG)		Hypothetical NADH pyrophosphatase	Inorganic ion transport and metabolism
699483	A	M	A	*rlpA*	SL1344_0626	699240..700307	−	A(GCT)		Rare lipoprotein A, role in cell division, contains SPOR domain, may bind peptidoglycan	
2733943	T	Y	T		SL1344_2552	2732413..2734785	−	A(GCA)		Bacteriophage SLP272, tail fiber- like protein	Bacteriophage
**NON-CODING**											
4372550	A	T	A	−	rRNA	3588585.. 3591592	−			23S ribosomal RNA	Transcription, ribosomal structure and translation
4415836	G	A	G	−	rRNA	4372578..4374122	+			16S ribosomal RNA	Transcription, ribosomal structure and translation
1282541	A	R	A	STnc150	SL1344_ncRNA_25	1282521..1282678	−			Experimentally verified small RNA (Sittka et al., 2008)	Regulation
3099732	G	T	G								

* Nucleotide denoted by S is either C or G; R is either G or A; K is either T or G; M is either A of C; Y is either T or C.

**Table:** 

**(B)**										
**Position**	**Nucleotide**			**Gene name**	**Reference ID**	**Location**	**Stand**	**Description**	**Function**	**Functional category**
	**SL1344**	**ST24^WT^**	**ST24^CHX^**							
**INTERGENIC**											
274042	C	C	M	−	SL1344_0700	Intergenic, pseudogene (783428..784579)	+	Pseudogene	UDP-galactopyranose mutase, involved in outer membrane biogenesis	
3708204	C	S	C	*glpR*	SL1344_3489	Intergenic, pseudogene (3707770..3708527)	−	Pseudogene	Hypothetical repressor of the glpD, glpFK, glpTQ, and glpACB operons involved in glycerol-3-phosphate metabolism	Anaerobic metabolism, lipid metabolism
4217516	A	G	A	−		Intergenic			182 bp up-stream of 16S ribosomal RNA (4416018..4417560)	
3485325	A	A	M	−		Intergenic			98 bp up-stream of 16S ribosomal RNA (4217614..4219158)	
3485327	A	A	W	−		Intergenic			35 bp up-stream of SL1344_3266 (folP), dihydropteroate synthase, involved in folate biosynthesis (- strand).	
783643	T	T	K	−		Intergenic			37 bp up-stream of SL1344_3266 (folP), dihydropteroate synthase, involved in folate biosynthesis (- strand)	
3848707	A	W	A	−		Intergenic			28 bp up-stream of SL1344_3605 (lpfB), fimbrial chaperone protein (- strand)	
1549186	A	W	A	−		Intergenic			1000 bp up-stream of SL1344_1445 (ydeI), hypothetical protein of unknown function	
1180820	A	R	A	−		Intergenic			385 bp up-stream from SL1344_1072 (ycdW), hypothetical 2-hydroxyacid dehydrogenase	
2073115	T	T	G	−		Intergenic			Bacteriophage SL203. Within a DNA invertase fragment	Bacteriophage
2045028	G	A	G	−		Intergenic			Bacteriophage SLP203	Bacteriophage
2762013	T	C	T	−		Intergenic			Bacteriophage SLP203	Bacteriophage
2762182	A	G	A	−		Intergenic			Bacteriophage SLP272	Bacteriophage
1282534	T	W	T	−		Intergenic			Bacteriophage SLP272	Bacteriophage
3117665	T	A	T	−	SL1344 repeat region 2	Intergenic, repeat region (3099171..3100233)	+	Repeat region	CRISPR repeat region	Bacteriophage
3117692	T	A	T	−	SL1344 repeat region 3	Intergenic, repeat region (3116271..3117792)	+	Repeat region	CRISPR repeat region	Bacteriophage
3435062	G	G	R	−	SL1344 repeat region 3	3116271..3117792	+	Repeat region	CRISPR repeat region	Bacteriophage
3590829	G	A	G	−	SL1344 repeat region 10	3435027..3435149	+	Repeat region	CRISPR repeat region	Bacteriophage

^*^Nucleotide denoted by S is either C or G; R is either G or A; K is either T or G; M is either A of C; Y is either T or C; W is either T or A.

### RNA purification and quantification

RNA was purified from 2 ml of each mid-logarithmic phase culture (OD_610 nm_ = 0.6), without chlorhexidine treatment and following exposure to 0.5 × MIC of the reference strain, equivalent to 1 μg/ml chlorhexidine, for 30 min using an Ambion RiboPure Bacteria Kit (Life Technologies Corporation, Carlsbad, CA). RNA was purified in accordance with the manufacturer's instructions, with the exception that the final DNase treatment step was carried out twice for each sample. Purified RNA was quantified using a ND-1000 spectrophotometer (Nanodrop, Thermo Scientific, Waltham, Massachusetts), and the quality of the RNA was assessed using a 2100 Bioanalyser (Agilent, Waldbronn, Germany).

### Transcriptomic analysis of purified RNA

**Figure 1:**
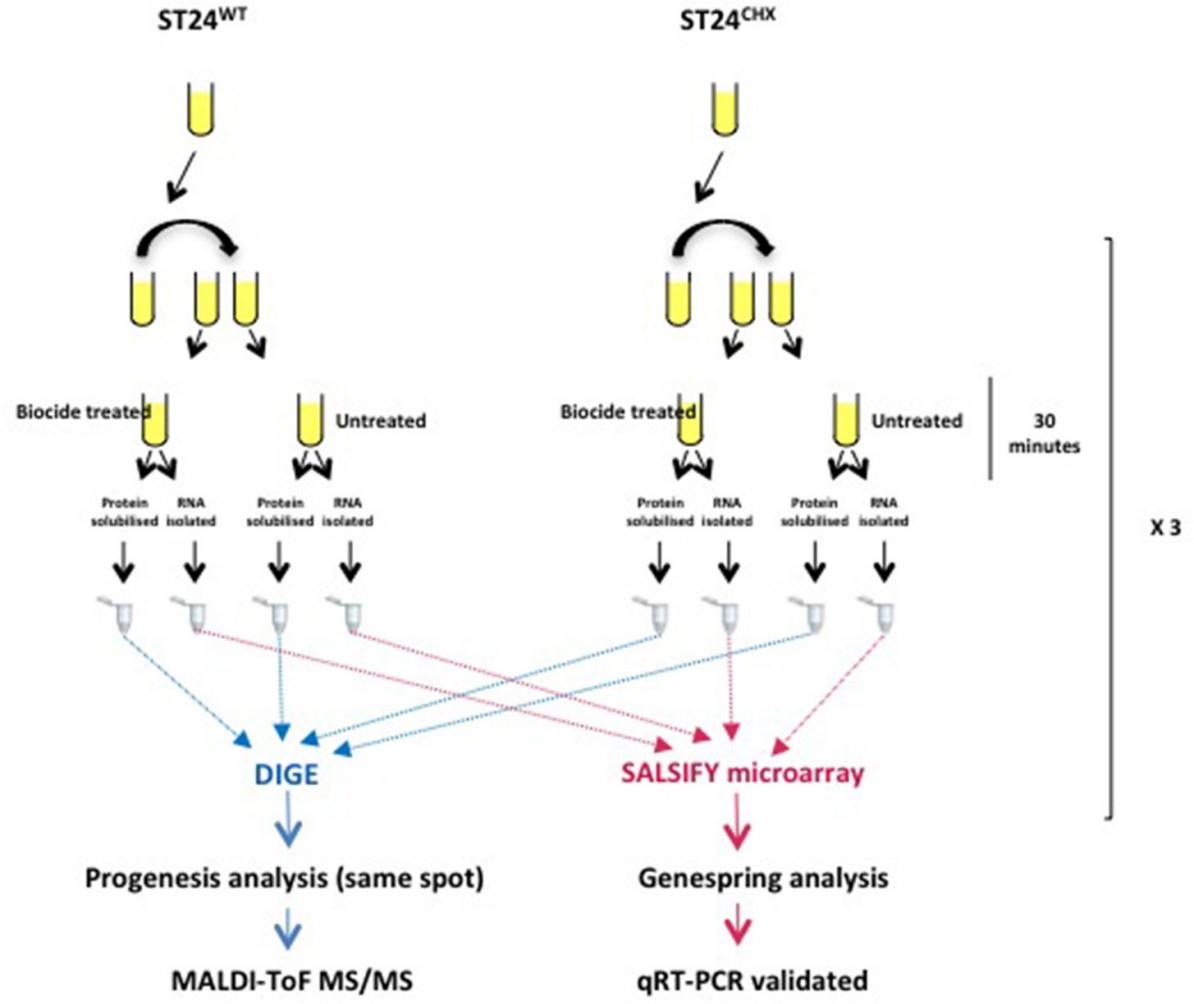
**Proteomic and transcriptomic experimental design for the analysis of differentially expressed genes and proteins comparing the reference chlorhexidine susceptible *Salmonella* Typhimurium, ST24^WT^ and its, isogenic, chlorhexidine tolerant mutant, ST24^CHX^**.

Conversion to and labeling of cDNA and hybridizations using SALSIFY microarrays (Design ID 026881) (Agilent technologies, Santa Clara, California), were carried out as described previously (Ygberg et al., 2006; Finn et al., 2013). In brief, RNA was converted to cDNA and fluorescently labeled with Cy3-dCTP using random priming. Genomic DNA was isolated from ST24^WT^ as described above. The genomic DNA (gDNA) was then labeled with Cy5-dCTP as a reference source. The labeled cDNA and gDNA were combined, denatured and hybridized to the microarray. Subsequently, the hybridization was carried out for 16–18 h at 65°C. The microarray slides were then washed according to manufacturer's instructions (http://www.chem.agilent.com/Library/usermanuals/Public/G2534-90004_HybridizationChamber_User.pdf). Microarray slides were cleaned with inert gas to remove any debris before scanning with the Agilent Microarray scanner system (Agilent technologies, Santa Clara, California). Scans were carried out at 5 μm resolution with Green and Red PMT values set to 100 % and an XDR value of 0.1. Images generated were saved as multi-image.tiff files. Feature extraction software (Agilent Technologies, Santa Clara, California) was used to extract the data. Hybridizations were repeated on three biological replicates (Figure 1).

The microarray transcriptional data were analyzed by using both GeneSpring GX software version 7.3 (Agilent Technologies, Santa Clara, California) and using R 2.14.2 (R Core Team, 2011), with base package statistics and the attached package Limma (version 3.10.2) (Smyth and Speed, 2003; Smyth, 2005; Ritchie et al., 2007). Genes with a fold change >2 and *p* < 0.05 were considered to be differentially expressed. Only those genes confirmed as differentially expressed by both the GeneSpring and R analytical methods, and detected by more than one oligonucleotide probe, were considered as significant, and are discussed further below. A summary of the statistical and expression data for all individual differentially expressed genes in provided in Table S1. Data from this study has been deposited in NCBI's Gene Expression Omnibus (GEO accession number GSE59566).

### qRT-PCR

Quantitative reverse transcriptase (RT)-mediated PCR (qRT-PCR) was performed on five selected gene targets, using specific primer pairs for three biological replicates (Table S2). Targets for validations were chosen as representative of the functional categories determined as differentially expressed from the microarray results, as well as one target non-differentially expressed. Bacterial cells were cultured to mid-logarithmic phase and total RNA was purified and quantified as outlined above. The levels of each RNA transcript were determined using a real-time *One-Step* RT-PCR assay (Eppendorf, Hamburg, Germany) with a QuantiTect SYBR Green RT-PCR kit (Qiagen, Hilden, Germany). The RT-PCR reaction was carried out in 25 μl volumes consisting of 12.5 μl 2 X QuantiTect SYBR Green RT-PCR Master Mix, 10 pmol of each primer pair, 32 ng of the purified RNA sample and 0.25 μl of QuantiTect RT Mix. RT-negative controls were included for each RNA sample to confirm that each was devoid of contaminating gDNA. A “no template control” was also included to ensure the reaction was free from contaminating RNA. Each qRT-PCR cycle consisted of an initial reverse transcription step of 30 min at 50°C followed by incubation at 95°C for 15 min. This latter step was immediately followed by 40 cycles of amplification consisting of; 15 s at 94°C, 30 s at 60°C and 30 s at 72°C. At the end of the 40 cycles the temperature was raised from 72 to 95°C, slowly, over 20 min. A final melt step, of 15 s at 95°C, ended the amplification programme. Relative gene expression was determined using the ΔΔC_T_ method. Two housekeeping genes were selected for this purpose and these included the DNA-directed RNA polymerase subunit B gene (*rpoB*) and the DNA gyrase subunit A gene (*gyrA*). A summary of all qRT-PCR data is provided in Table S3.

### Fluorescent differential gel electrophoresis (DIGE) analysis of proteins

For proteomic analysis, cell pellets were prepared from the 25 ml volumes of the mid-logarithmic phase cultures (OD_610 nm_ = 0.6) by centrifugation at 3220 × g for 10 min. The resulting cell pellets were maintained on ice and washed in 1 ml ice-cold 10 mM Tris-Cl [pH 8], 1 mM EDTA (TE buffer) three times. The washed cell pellet was then resuspended in 200 μl solubilization buffer (7 M urea, 2 M thiourea, 1% ASB-14) for 18 h at room temperature. Proteins were quantified using the RCDC Protein Assay (Bio-Rad, Hercules, California) according to the manufacturer's instructions.

All samples were diluted to a final protein concentration of 5 μg/ml and adjusted to pH 8.5 using 10 mM NaOH. A pooled internal standard comprising 33.33 μg of each sample was labeled with Cy2 dye (GE Healthcare, Buckinghamshire, UK) following the manufacturer's instructions. For each sample, 60 μg of protein was labeled with Cy3 or Cy5 (GE Healthcare, Buckinghamshire, UK), to control for dye swap (Figure 1).

Samples were then pooled as follows; 50 μg each of the reference strain ST24^WT^ samples (either Cy3 or Cy5 labeled) were combined with 50 μg of the corresponding mutant ST24^CHX^ sample (labeled with the alternative dye). This was followed by the addition of 50 μg of internal standard. Pooled protein samples were then diluted to 450 μl in solubilization buffer (30 mM DTT, 0.5% IPG buffer [GE healthcare, Buckinghamshire, UK] and 0.01% bromophenol blue) for first dimension separation.

First dimension separation was carried out using Immobiline DryStrip gels (IPG strips) (GE healthcare, Buckinghamshire, UK) as previously described (Nally et al., 2005, 2007). In brief, 24 cm pH 4–7 IPG strips were rehydrated with protein samples overnight, as per the manufacturer's instructions. Isoelectric focusing was carried out using the Ettan IPGphor II Isoelectric focusing system (GE Healthcare, Buckinghamshire, UK) with the following parameters: Step and hold (Sth) 3500 V for 75,000 V/h, Gradient 8000 V for 10 min, Sth 8000 V for 1 h, Sth 100 V for 5 h. At the end of the isoeletric focusing step, strips were equilibrated for 10 min in equilibration buffer (6M urea, 0.75 M Tris-Cl pH 8.8, 29.3% (w/w) glycerol, 1% (w/v) SDS) containing 1% (w/v) DTT with gentle shaking at 80 rpm, followed by washing for 10 min in equilibration buffer containing 2.5% (w/v) iodoacetamide with shaking at 80 rpm. Strips were then loaded on a 12% (w/v) sodium dodecyl sulfate polyacrylamide gel electrophoresis (SDS-PAGE) gel for the second dimension separation. Gels were electrophoresed at 2 W (per gel) for 1 h, followed by 4 W (per gel) for 18 h and, finally, at 12 W (per gel) until the dye front reached the bottom of the gel on visual inspection using the Ettan DALT*six* Electrophoresis system. The entire process was carried out using low fluorescent glass plates and in the dark. Gels were subsequently imaged on a Typhoon™ variable mode imager (GE Healthcare, Buckinghamshire, UK) according to the manufacturer's instructions, and the output analyzed using Progenesis SameSpots software (^©^Nonlinear Dynamics Ltd., Newcastle, UK). Only *t*-test analysis spots with a *p* < 0.05, power >80% and *q* < 0.05 were considered differentially expressed. The experimental design is summarized in Figure 1. A summary of the statistical and expression data for all individual differentially expressed protein spots in provided in Table S4.

### Excision and digestion of spots of interest for identification by mass spectrometry (MS)

A 2-D SDS-PAGE master gel was electrophoresed on 24 cm IPG strips as described above, with the exception that only 400 μg of un-labeled internal standard was loaded. The second dimension separation was carried out as before, with the exception that glass plates were pre-treated for 1.5 h with a bind silane solution (80% ethanol, 2% glacial acetic acid, 0.1% bind saline) (GE Healthcare) and reference markers were attached as outlined in the manufacturer's instructions (GE Healthcare). The resolved proteins were visualized using SYPRO-Ruby stain (Sigma, Saint Louis, Missouri) as per manufacturer's instructions. The master gel was scanned using the Typhoon™ variable mode imager (GE Healthcare), and the output analyzed using Progenesis SameSpots software. A pick list was generated, which included the coordinates of both differentially expressed and non-differentially expressed spots on the master gel. The protein spots on the pick list were excised and digested using the Ettan Spot Handling Workstation (GE Healthcare), and the resulting peptide mixtures used for MS-based protein identification.

All MS and MS/MS analyses were performed using a 4800 MALDI TOF/TOF (Applied Biosystems, Foster City, CA, USA). For each sample one MS spectrum was acquired, and the eight most intense precursors were subsequently selected for MS/MS analysis (Sergeant et al., 2011). An Applied Biosystems GPS-server was used for database searches with an in-house MASCOT platform (Matrix Science, www.matrixscience.com, London, UK). The spectra from one spot (combined MS and eight MS/MS spectra) were submitted and compared against the NCBInr database limited to bacterial proteins only (downloaded from the NCBI server on 26/09/2011 containing 8,874,873 bacterial sequences). A mass window of 100 ppm for the precursor and 0.75 Da for the fragments was tolerated. During the database searches the following parameters were defined: two missed cleavages, fixed carbamidomethylation of cysteine, variable oxidation of methionine and tryptophan to kynurenine or double oxidation to *N*-formylkynurenine. All the identification data are included in Table S5. All identifications were manually validated, and extra precursors were selected for fragmentation if the data obtained was judged to be insufficient. The MS-spectra of spots wherein the same protein was identified were compared, and extra precursors (unique to one of the spectra) were fragmented to distinguish the molecular forms present in the individual spots (Carpentier et al., 2011).

### Phenotypic microarray assay

The chlorhexidine susceptible reference strain ST24^WT^ and chlorhexidine tolerant mutant ST24^CHX^ were examined for phenotypic divergence using Omnilog™ phenotypic microarrays (using all plates denoted PM 1 through 20) (BioLog Inc., Hayward, California). Bacterial cell suspensions were prepared and PM plates were inoculated following manufacturers' instructions. These plates were incubated at 37°C for 48 h in an Omnilog™ microplate reader (BioLog Inc., Hayward, California).

The digital imagery of this instrument tracks changes in the respiration of bacterial cultures growing in individual wells over time. The Omnilog™ output for a given plate consists of an optical density (OD) reading for each well, recorded every 15 min over the 48 h period. The data output for the reference and mutant strains were analyzed using Omnilog™ PM software, and negative controls (wells containing the inoculated Omnilog™ growth medium, but without any substrate, used to normalize differences in inoculums and redox dye oxidation between samples) were subtracted from each reading for each plate. The resulting kinetic profiles for ST24^WT^ and ST24^CHX^ were compared using an integration function. A divergent phenotype was identified when a difference in Omnilog™ units of 20,000 or greater between the strains was obtained following integrative function analysis. A summary of the differential phenotypes is provided in Table S6.

## Results and discussion

Chlorhexidine tolerance has been documented in several bacterial genera including *Pseudomonas, Klebsiella*, and *Serratia* (Nde et al., 2009). Bacteria demonstrating a reduced susceptibility to chlorhexidine can be selected *in vitro*, as in the case of *Salmonella* (Braoudaki and Hilton, 2004; Condell et al., 2012b), *Escherichia coli* (Braoudaki and Hilton, 2004) and *Staphylococcus aureus* (Kaatz et al., 2005; Huet et al., 2008). Based on our current understanding of the modes of action related to various biocides, tolerance to these compounds, including chlorhexidine, typically does not develop following mutation to a particular target gene but rather involves broader cellular changes, such as up-regulated efflux pump activity or alterations in cell wall permeability (Poole, 2002). In some instances, the tolerant phenotype that develops may correlate with a reduced susceptibility to other antimicrobial agents, for example, chlorhexidine exposure has been associated with a reduced susceptibility to clinically important antimicrobial agents (Braoudaki and Hilton, 2004; Condell et al., 2012a). In this study, our aim was to describe the genotype and phenotype(s) exhibited by *S.* Typhimurium following sub-lethal exposure to chlorhexidine. Two *S.* Typhimurium isolates, a chlorhexidine susceptible reference strain, denoted as ST24^WT^ and its isogenic chlorhexidine tolerant counterpart ST24^CHX^, were studied in detail. We explored whether or not tolerance to chlorhexidine was a multi-factorial process, and if such a phenotype evolves following one or more mutations in a target gene, as occurs with triclosan (Heath and Rock, 2000; Condell et al., 2012b; Sheridan et al., 2012).

To assist with a logical approach to describing the data from this study, the results and discussion are outlined as follows; firstly, the bacterial response of ST24^WT^ to chlorhexidine exposure is described. This section is followed by a description of the response of the chlorhexidine tolerant ST24^CHX^ following exposure to the biocide. Finally, a comparison of ST24^WT^ and ST24^CHX^ is shown and a general chlorhexidine response network in *Salmonella* Typhimurium based on these data is proposed.

### Response of *Salmonella* typhimurium ST24^WT^ to sub-lethal chlorhexidine exposure

Bacterial responses comparing *S.* Typhimurium ST24^WT^ in the absence of biocide and following a 30 min exposure to 0.5 × MIC of chlorhexidine was carried out. Figure 1 provides an overview of the experimental strategy used.

**Figure 2:**
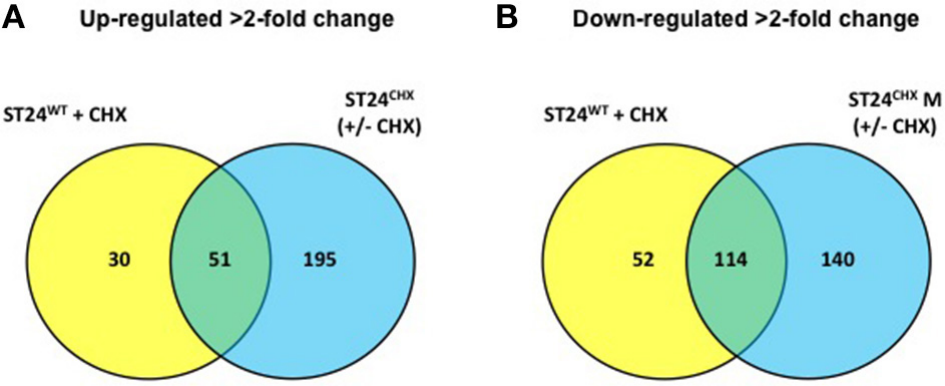
**Number and distribution of genes (A) up-regulated in the sensitive ST24^WT^ following chlorhexidine exposure and/or the tolerant mutant ST24^CHX^ relative to the reference strain (ST24^WT^) without chlorhexidine exposure. (B)** Down-regulated in the sensitive ST24^WT^ following chlorhexidine exposure and/or the tolerant mutant ST24^CHX^. The figure shows the differentially expressed genes relative to the reference strain (ST24^WT^) without chlorhexidine exposure.

**Figure 3:**
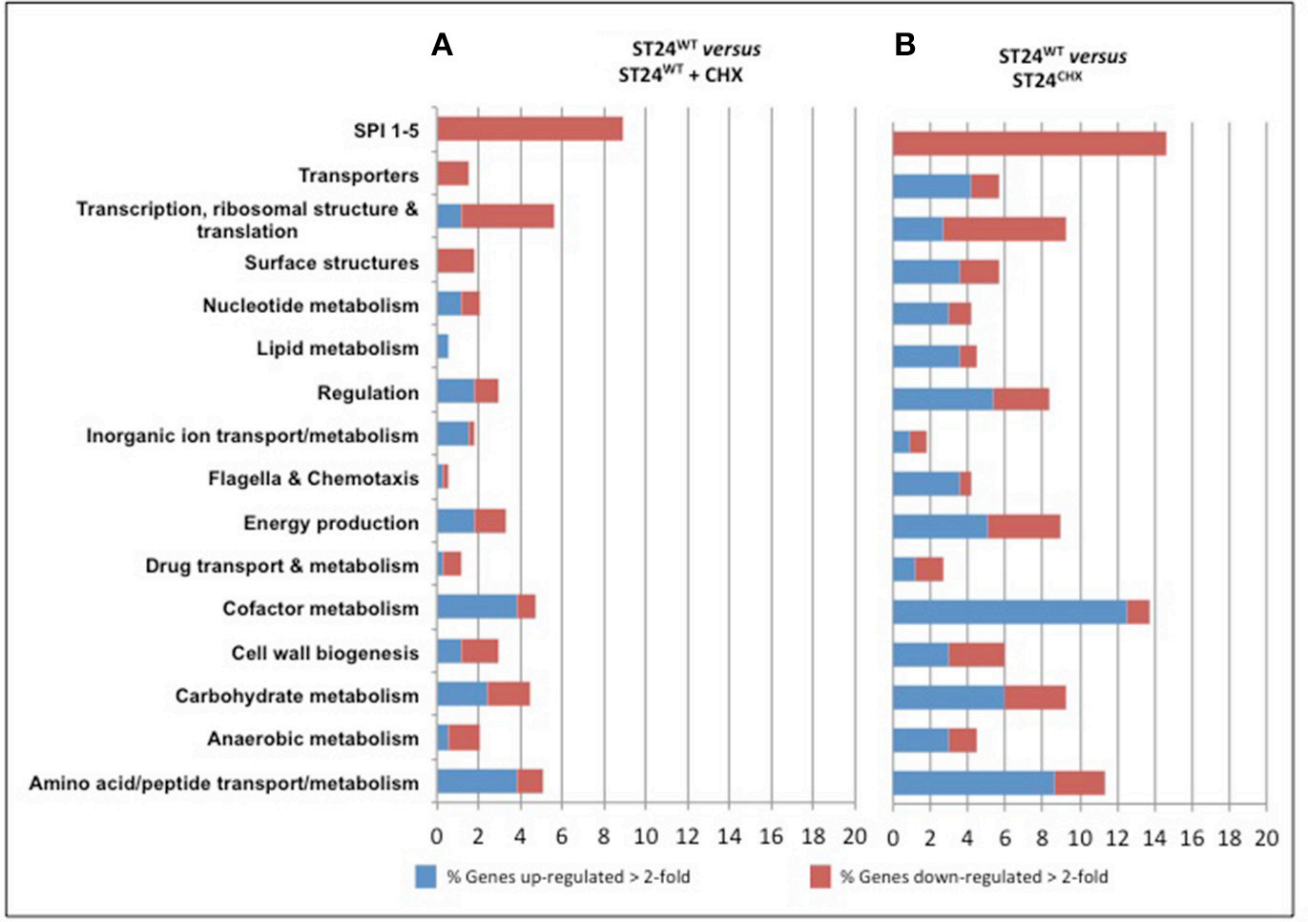
**Functional groups of genes differentially expressed between (A) the reference ST24^WT^ in the absence of chlorhexidine exposure and the reference isolate following chlorhexidine exposure and (B) the mutant isolate without chlorhexidine exposure**. The red and blue coloring indicates the percentage of those genes from each functional category that were differentially expressed. The numbers of genes included in the functional group analysis is given in Table S1. The lists of genes comprising the functional groups retrieved from the Kyoto Encyclopedia of Genes and Genomes (KEGG) (http://www.genome.jp/kegg/).

**Figure 4:**
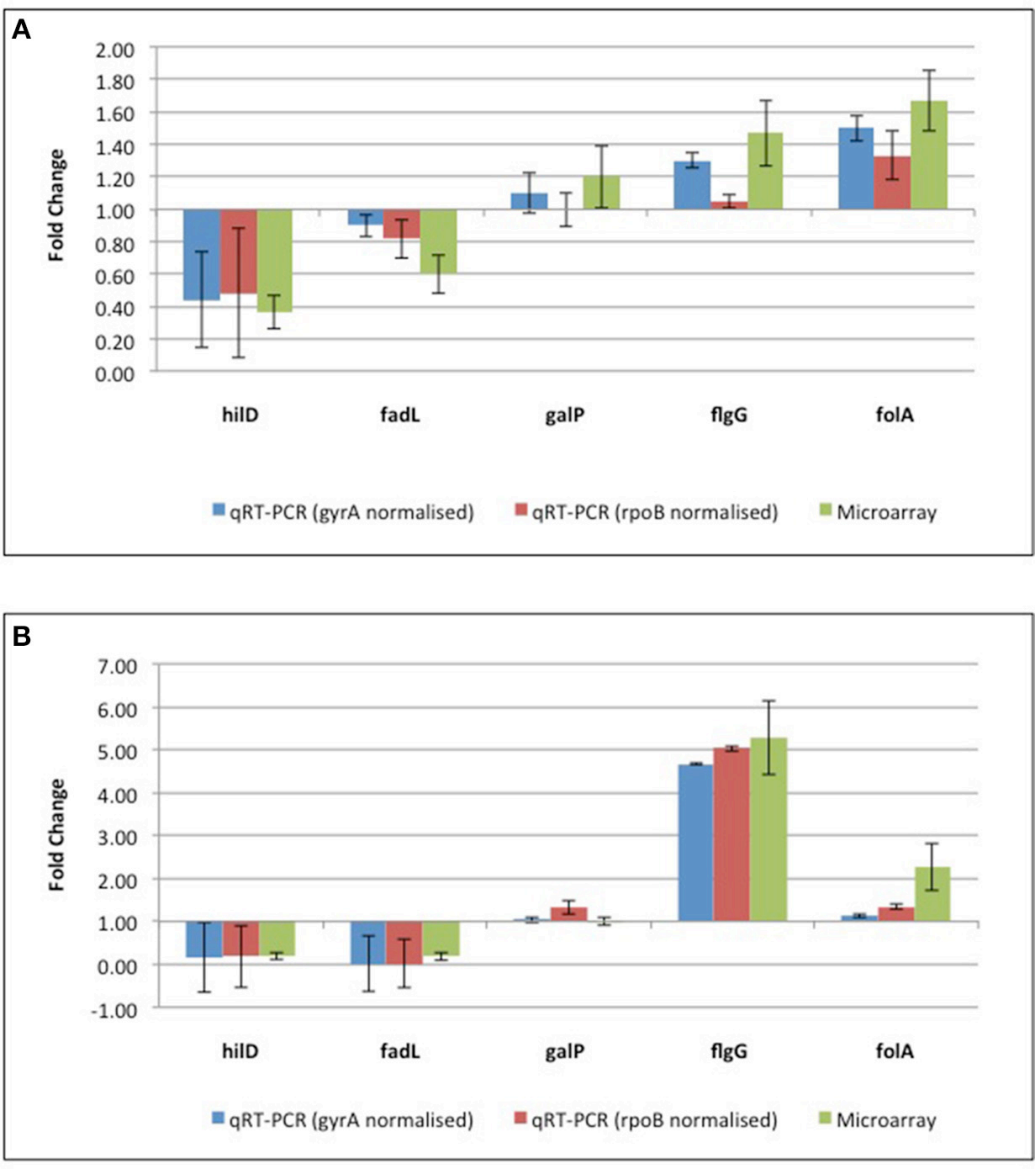
**qRT-PCR confirmation of microarray data from (A) the reference ST24^WT^ chlorhexidine treated, relative to the same isolate in the absence of chlorhexidine exposure and (B) the mutant isolate, ST24^CHX^, relative to the reference ST24^WT^**. Details of protein results are provided in Table S4, with qRT-PCR and transcriptomic results shown in Table S3.

A total of 247 genes were significantly differentially expressed (2-fold-change, *p* < 0.05), following sub-lethal exposure to chlorhexidine (Table S1); 81 of these genes were up-regulated whilst 166 were down-regulated (Figure 2). Differentially expressed genes of known function were assigned to functional categories (Figure 3 and Table S1). Transcriptomic data were subsequently validated using qRT-PCR for 5 selected genes. Similar fold-change values were obtained from both independent analyses, for all five genes (Figure 4A, Table S3). Results from transcriptomic profiling revealed that the functional categories containing the largest number of up-regulated genes were involved in general cell metabolism; amino acid transport/metabolism, cofactor metabolism and carbohydrate metabolism. Conversely, the functional groups containing the largest number of down-regulated genes included virulence, transcription, translation and ribosomal structure, along with phage-associated genes. For each functional category, the numbers of differentially expressed genes are shown as a percentage of the total number of genes in that category (as determined by KEGG, Figure 3). Corresponding gene lists are provided in Table S1.

DIGE analysis compared the proteome of the susceptible ST24^WT^ reference strain in the absence and presence of chlorhexidine. A total of 1431 protein spots were aligned by Progenesis SameSpots software. No differentially expressed proteins were detected when the proteomes of ST24^WT^ was compared before and after chlorhexidine exposure for 30 min. This contrasts to the response of *S*. Typhimurium following the treatment to other biocidal agents. Numerous differentially expressed proteins were apparent in *S*. Typhimurium following 30 min exposure to the biocide triclosan, a similar response was also noted in *E. coli* following exposure to the same agent (Condell et al., 2012b; Sheridan et al., 2012).

### General cell metabolism of ST24^WT^ following sub-lethal exposure to chlorhexidine

**Figure 5:**
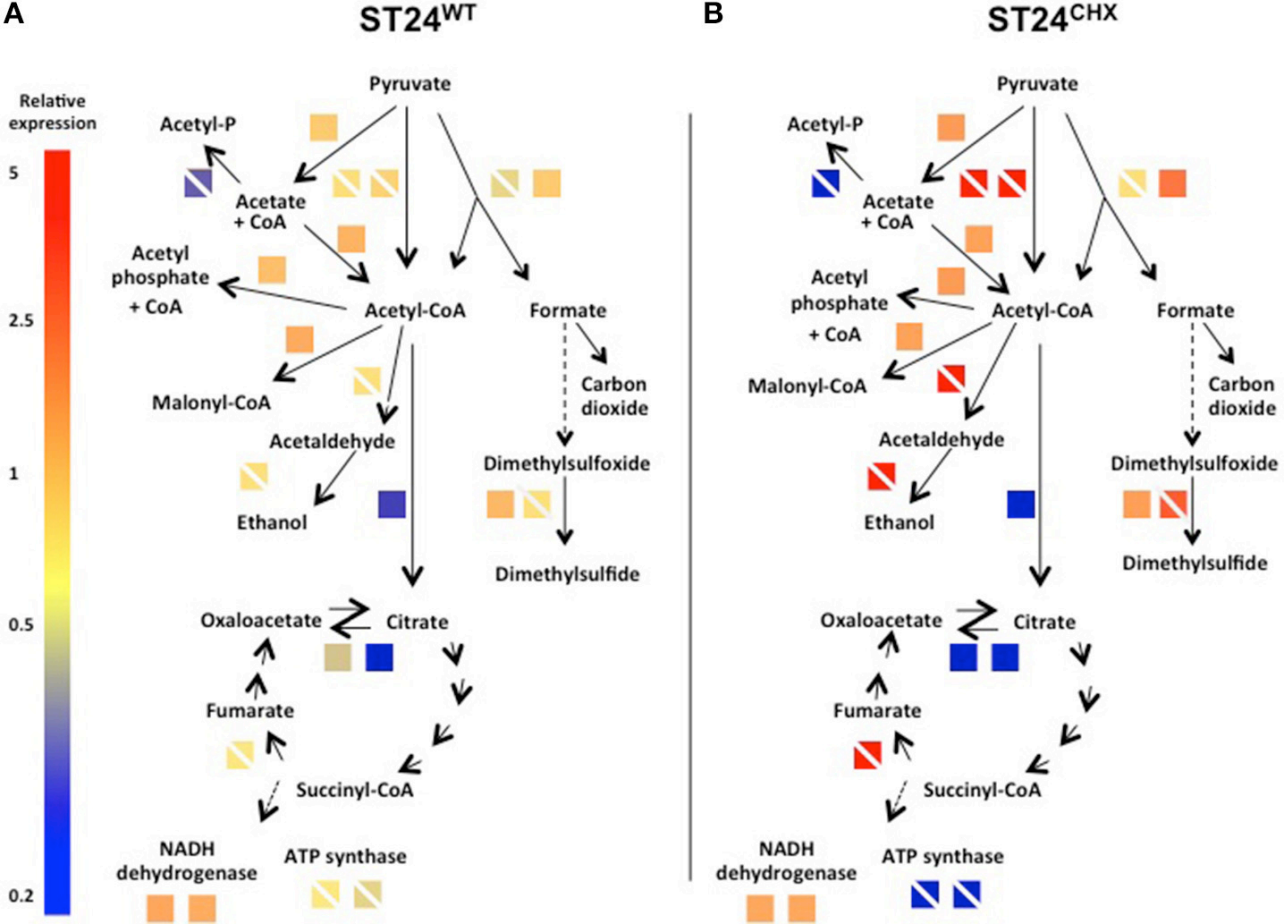
**Expression of differentially expressed genes and/or proteins associated with anaerobic metabolism/energy production in (A) the reference isolate ST24^WT^ following chlorhexidine exposure and (B) the mutant isolate ST24^CHX^ compared to its isogenic ST24^WT^ without chlorhexidine exposure**. Enzyme names, descriptions, gene identifiers and fold-change values are given in Table 2, Tables S1, S4. Color blocks without a white diagonal line represent gene fold change values (Table S1), color blocks containing a white diagonal line represent protein fold change values (Table 2, Table S4).

When analyzed using the transcriptomic platform, genes associated with central metabolism and energy production in ST24^WT^ exhibited altered expression. Enzymes associated with glycolysis and the TCA cycle were down-regulated, whilst those of other alternative pathways, such as mixed acid fermentation, fatty acid biosynthesis and glyceroplipid metabolism were up-regulated (Figure 5A). The expression of formate dehydrogenase was increased, and this enzyme functions in the conversion of formate to CO_2_ in the absence of alternative electron acceptors. In addition, acetyl-CoA carboxylase, an enzyme that converts acetyl-CoA to malonyl-CoA, a regulatory step for the inititation of fatty acid biosynthesis, was also up-regulated. Genes associated with anaerobic glycerolipid metabolism, such as *glyB*, were up-regulated in ST24^WT^. Interestingly, genes encoded by the *pdu* operon, which is associated with the catabolism of 1,2-propanediol and linked with the formation of polyhedral bodies- large proteinaceous structures of unknown function (Bobik et al., 1999; Havemann et al., 2002), were up-regulated. This metabolic pathway is known to be involved with anoxic metabolism in *Salmonella* (Bobik et al., 1999; Havemann et al., 2002), and it has previously been proposed that chlorhexidine blocks oxygen utilization in bacteria (Barrett-Bee et al., 1994). ST24^WT^ appeared to up-regulate alternative metabolic pathways for anoxic energy production to mitigate this inhibitory action of chlorhexidine (Figure 5A).

### Cofactor metabolism of ST24^WT^ following sub-lethal exposure to chlorhexidine

**Figure 6:**
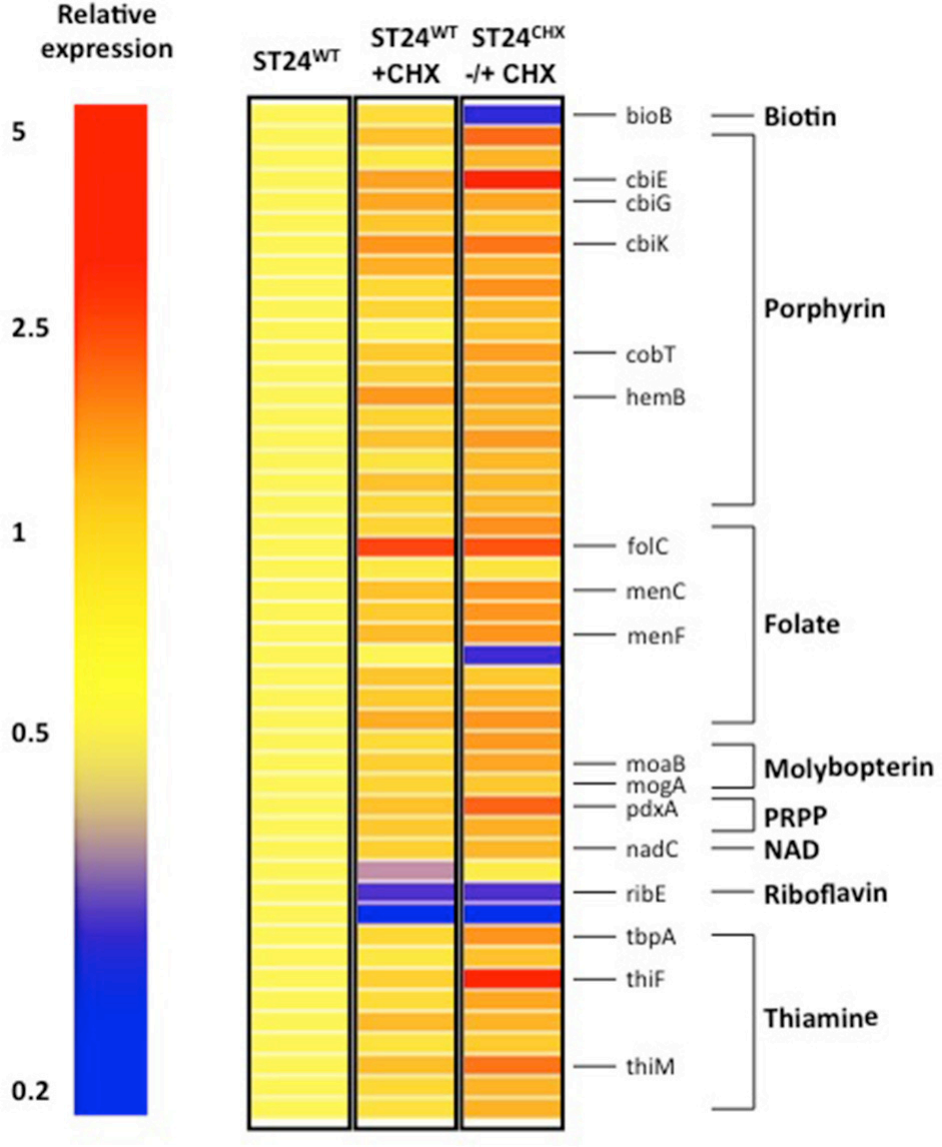
**Chlorhexidine tolerance is associated with differentially expressed genes associated with cofactor metabolism**. Relative expression refers to the fold-change difference in expression between the ST24^WT^, without chlorhexidine treatment relative to the conditions listed in the figure.

In addition to alterations in the general cell metabolism, genes associated with cofactor synthesis in ST24^WT^ were differentially-expressed. Six % of genes known to be associated with cofactor biosynthesis (as obtained from KEGG) were up-regulated in ST24^WT^, following a 30 min exposure to chlorhexidine. These markers coded for enzymes involved in the synthesis of thiamine, folate, molybdopterin and porphyrin (vitamin B_12_) amongst others (Figure 6). The altered expression of these cofactor associated genes may be linked with the changes in general cell metabolism outlined earlier. For example, the increased expression detected in enzymes involved in both the synthesis and transport of thiamine may be a bacterial response designed to satisfy the increased demand for thiamine as a cofactor for pyruvate dehydrogenase and 2-oxoglutarate decarboxylase. Similarly, vitamin B_12_ is required for the utilization of 1,2-propanediol as a carbon source by the *pdu* operon and molybopterin as a cofactor of formate dehydrogenase. All were up-regulated in ST24^WT^, following exposure to chlorhexidine (Gladyshev et al., 1994; Bobik et al., 1999).

### Modifications in membrane structural and transport systems of ST24^WT^ following sub-lethal exposure to chlorhexidine

Reduced susceptibility to chlorhexidine associated with modifications in the cell wall/ cell membrane has been reported previously (Russell and Furr, 1986; Russell and Path, 1986; Tattawasart et al., 2000). As an initial step in forming contact with the bacterial cell, due to its positive charge, chlorhexidine is thought to associate with negatively-charged residues on the LPS structures of Gram-negative bacteria (Tattawasart et al., 2000). Modifications of the LPS structure, such as those that contribute to reducing its negative charge along with a reduction in the O-antigen polymer are associated with a decrease in susceptibility to cationic agents (Tattawasart et al., 2000).

In this study, several changes in the expression of genes associated with cellular permeability were noted in ST24^WT^ following chlorhexidine exposure. An increase in the expression of genes associated with the biosynthesis of the peptidoglycan subunits (*murG*) and responsible for the cross-linking between these subunits (*murD, murG*) was noted. Up-regulation of these genes may provide a protective modification that contributes to an increase in thickness and/or the cross-linking of the peptidoglycan layer, changes that would affect the efficacy of antimicrobial agent action and/or transport (Gilbert and Brown, 1980; McDonnell and Russell, 1999). Additionally, ST24^WT^ exhibited a down-regulation in genes of the *rfb* locus, involved in O-antigen synthesis; namely *rfbU* and *rfbA*. The O-antigen provides several anionic binding sites (Zorko and Jerala, 2008) and modification may result in altered chlorhexidine binding, thereby reducing the permeability of the cell to this agent. Modifications in O-antigen structure have previously been associated with an increased tolerance to clinically important cationic antimicrobials in *Pseudomonas* (Bryan et al., 1984; Hasegawa et al., 1997; Tattawasart et al., 2000).

### Efflux pump activity of ST24^WT^ following sub-lethal exposure to chlorhexidine

It has previously been reported that an increase in the expression of certain efflux systems is correlated with a reduction in the susceptibility to chlorhexidine in several bacterial species studied, including *P*. *aeruginosa* (mediated through MexCD–OprJ), *S*. *aureus* (QacAM MepA) and *E. coli* (AcrAB-TolC) (Brown and Skurray, 2001; Levy, 2002; Poole, 2004; Huet et al., 2008). However, in this study the reference isolate ST24^WT^ showed no increase in the expression of any efflux systems following chlorhexidine exposure for 30 min. However, a decrease in the expression of AcrE, a component of the AcrEF-TolC efflux system and the multi-drug efflux associated gene *ydhE*, a member of the MATE super-family (Omote et al., 2006) were observed. From the study reported by Gilbert and Moore, the over-expression of efflux pumps do not reduce the antimicrobial effect of chlorhexidine, as the biocide does not become solubilized in the bacterial membrane core (Gilbert and Moore, 2005). Thus, the contribution of these efflux systems in terms of altered chlorhexidine susceptibility requires further investigation.

### SOS response of ST24^WT^ following sub-lethal exposure to chlorhexidine

Twelve genes encoding DNA binding proteins (other than transcriptional regulators) or associated with DNA damage/repair mechanisms were up-regulated (Table S1). Following exposure to chlorhexidine, ST24^WT^ showed an increase, in four genes associated with the SOS response (Table S1). Recent work has suggested DNA damage as a means by which chlorhexidine inactivates periodontal bacteria (Yeung et al., 2007; Nde et al., 2009). It was reported that chlorhexidine can cause DNA-DNA cross-linking, thereby interfering with DNA metabolism and leading to DNA strand breaks (Yeung et al., 2007; Nde et al., 2009). If the same mechanism holds true for *Salmonella*, then the up-regulation in the DNA repair systems along with DNA binding proteins might represent the bacterial response to mitigate the DNA cross-linking effects of chlorhexidine. A similar feature was reported earlier in *E. coli* (Allen et al., 2006)

### Transcription and translation in ST24^WT^ following sub-lethal exposure to chlorhexidine

Several genes encoding proteins associated with transcription and translation were down-regulated following chlorhexidine exposure (Figure 3). A decrease in the expression of the α-sub-unit of DNA-directed RNA polymerase (*rpoA*) was detected. Furthermore, there was a down-regulation in 11 genes coding for ribosomal subunits, including multiple 30S and 50S ribosomal sub-unit components, at the RNA level. Chlorhexidine has previously been associated with a decrease in protein synthesis (Galice et al., 2006; Nde et al., 2009).

### Virulence gene expression in ST24^WT^ following sub-lethal exposure to chlorhexidine

**Figure 7:**
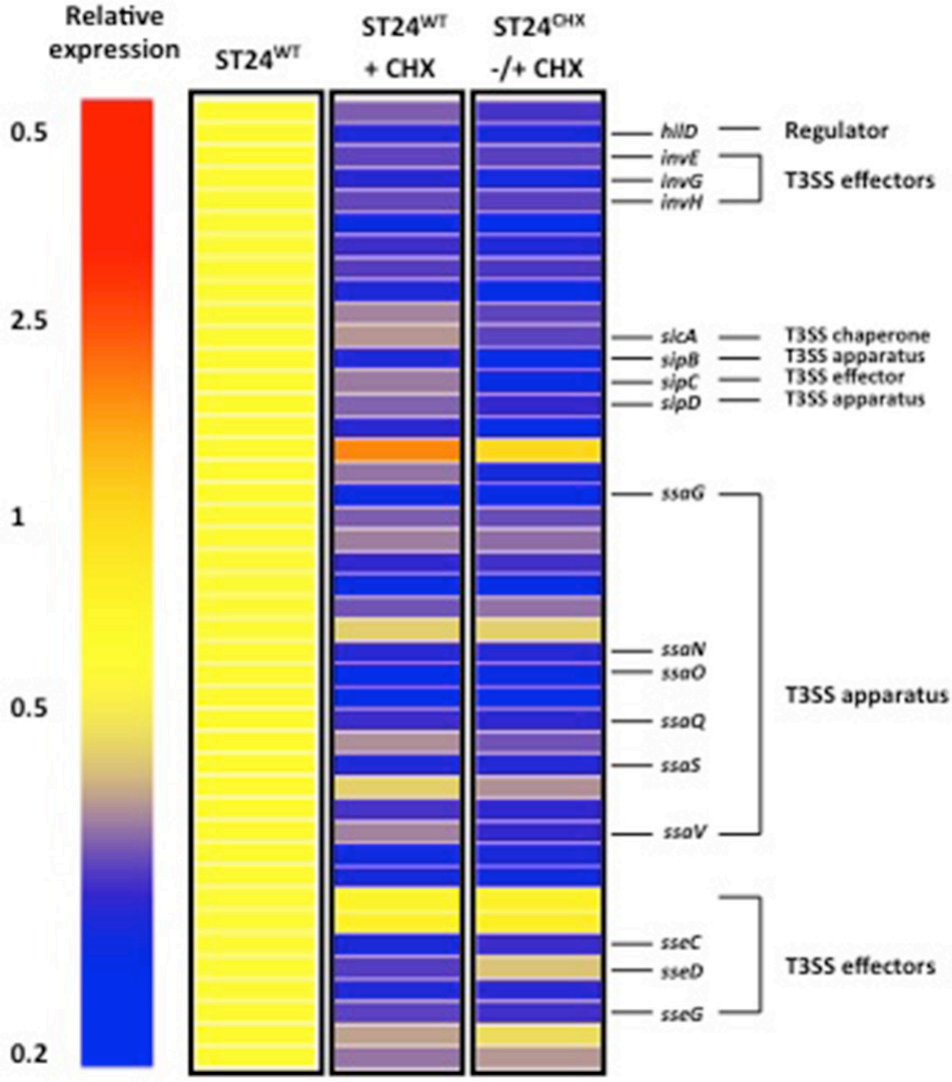
**Chlorhexidine tolerance is associated with differential expression of SPI-1 and SPI-2 virulence genes**. Relative expression refers to the fold-change in expression between the reference ST24^WT^, without chlorhexidine treatment relative to the conditions listed in the figure.

Transcriptomic profiling of the chlorhexidine susceptible ST24^WT^ revealed a correlation between chlorhexidine exposure and a reduction in the expression of virulence associated genes (Figure 7). A total of 8% of the genes encoded by SPI-1, SPI-2, SPI-3, SPI-4, and SPI-5 (obtained from KEGG) were down-regulated following chlorhexidine exposure in ST24^WT^. Studies examining the association between chlorhexidine tolerance and alterations in virulence phenotypes are lacking in the literature. It has been reported that reduced chlorhexidine susceptibility in a collection of β-haemolytic *E. coli* correlated with four virulence factor genotypes (Beier et al., 2005). However, a reduction in virulence factor expression in *Streptococcus agalactiae* exposed to sub-inhibitory concentrations of chlorhexidine was reported (Galice et al., 2006), while sub-lethal concentrations resulted in a significant reduction in infectivity of mice by *E. coli* and *Klebsiella aerogenes* (Holloway et al., 1986). This reduction in virulence following chlorhexidine exposure may contribute to its potency as a therapeutic antimicrobial agent.

### Response of *Salmonella* typhimurium ST24^CHX^ to sub-lethal chlorhexidine exposure

The reference isolate, ST24^WT^ was exposed to several rounds of *in vitro* selection using sub-lethal concentrations of chlorhexidine, as described previously by Condell et al. (2012a). A *S.* Typhimurium isolate denoted as ST24^CHX^ was recovered which exhibited a high-level chlorhexidine tolerance phenotype, with a >50-fold increase in the MIC in comparison to ST24^WT^ (Condell et al., 2012a). The transcriptomic and proteomic responses of ST24^CHX^ were studied following a 30-min exposure to this biocide, in the same way as described for the wild-type isolate. Alterations in the bacterial response were compared to those detected for ST24^CHX^ without biocide exposure.

Exposure of ST24^CHX^ to chlorhexidine did not cause differential expression of any genes. Similarly, no differentially expressed proteins were detected. We conclude that exposure to 0.5 × MIC chlorhexidine for 30 min did not affect the transcription or translation of any genes in the tolerant mutant.

### General comparisons of SNPs, gene and protein expression in ST24^WT^ and ST24^CHX^

The mutant (ST24^CHX^) was studied in detail, using several analytical approaches to identify cellular mechanisms associated with the tolerance to chlorhexidine. Results from these analyses were compared to those from the reference susceptible isolate (ST24^WT^), described above, to identify any changes of interest.

Sixty SNPs that distinguished ST24^CHX^ from its isogenic chlorhexidine susceptible reference ST24^WT^ were identified. Of these, 18 SNPs were located in intergenic regions and 42 SNPs occurred within genes. The intergenic SNPs included 5 located within bacteriophage regions (annotated as L203 or SLP22), 4 located within clustered regularly interspaced short palindromic repeat (CRISPR) regions and 2 identified within pseudogenes. Of the SNPs that occurred within genes, 17 were synonymous, 15 were non-synonymous, 6 were degenerative and 4 were within non-coding genes. The non-synonymous and degenerative polymorphisms occurred within genes associated with bacteriophage, drug transport, virulence, transport/permeability and general cell metabolism, whilst the non-coding SNPs were within sRNA and ribosomal RNA regions. A summary of all SNPs identified between the reference and the mutant isolates are given in Table 1.

Differentially expressed transcripts were identified when comparing the transcriptomes of ST24^WT^ and ST24^CHX^, without chlorhexidine exposure. A total of 500 genes were differentially expressed between both isolates (Table S1), with 246 of these being up-regulated and 254 down-regulated (Figure 2). Of these differentially expressed genes, those of known function were assigned into functional categories (Figure 3 and Table S1). As before, functional categories containing the largest number of up-regulated genes included general cell metabolism; amino acid, cofactor and carbohydrate transport/metabolism. Conversely, the functional groups containing the largest number of down-regulated genes included virulence, transcription, translation and ribosomal structure, along with phage-associated genes. Gene lists of the differentially expressed transcripts and their functional categories are provided in Table S1. A qRT-PCR assay was performed on 5 selected genes, and similar fold changes were obtained from both independent analyses for all of the chosen genes (Figure 4B). A summary of all qRT-PCR data are provided in Table S3.

**Figure 8:**
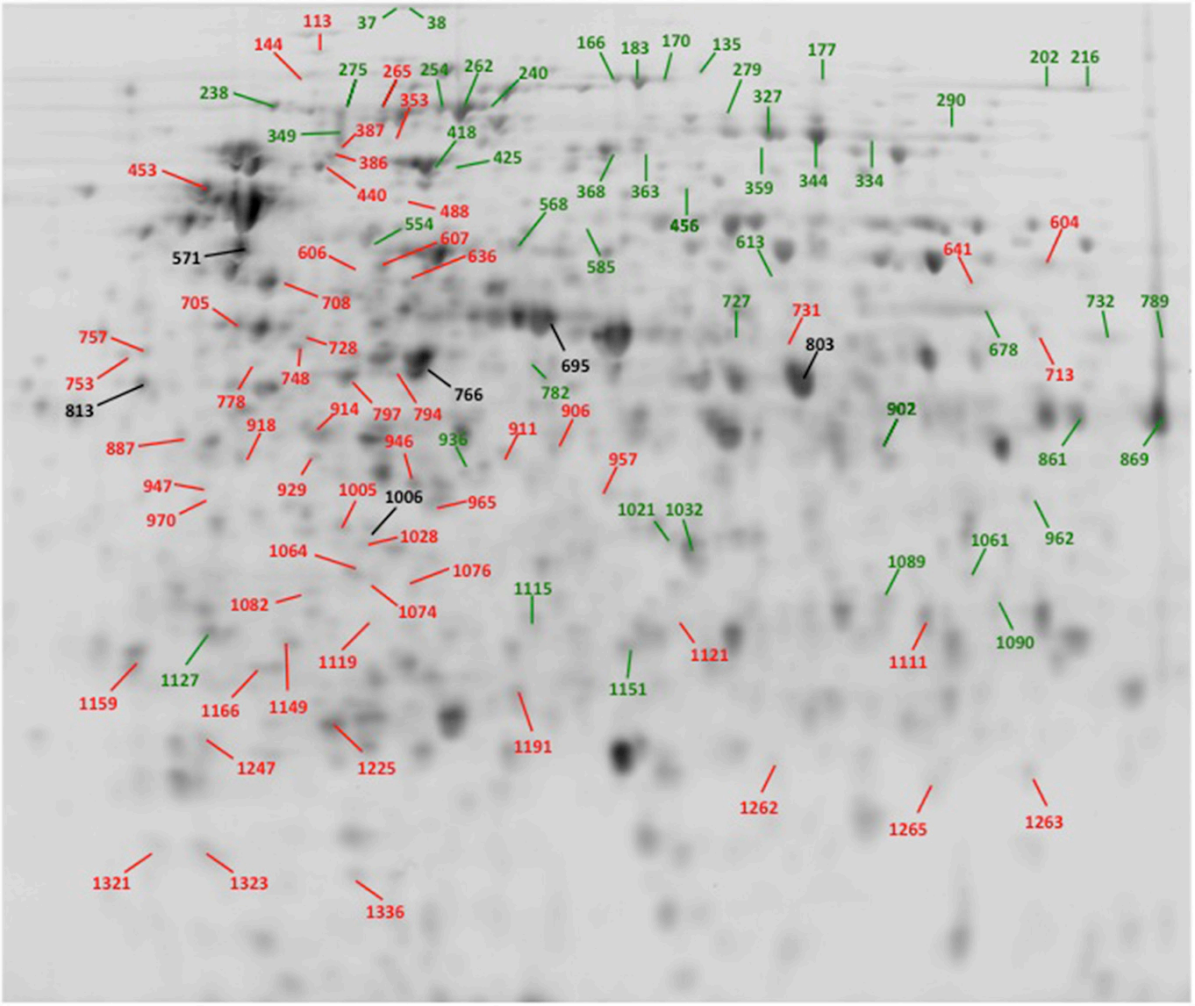
**A 2-D proteome map of ST24^WT^ and ST24^CHX^, without chlorhexidine treatment**. The spot numbers corresponds to identified proteins as described in Table 2, Tables S4, S5. Those spots labeled in black represent non-differentially expressed proteins (see Table S5). Protein spots labeled green (and denoted as Group A, see Table 2, Table S4) were significantly up-regulated in ST24^CHX^ relative to ST24^WT^, whilst spots labeled in red represent Group B and were significantly down-regulated in ST24^CHX^ relative to ST24^WT^ (Table 2, Table S4).

**Figure 9:**
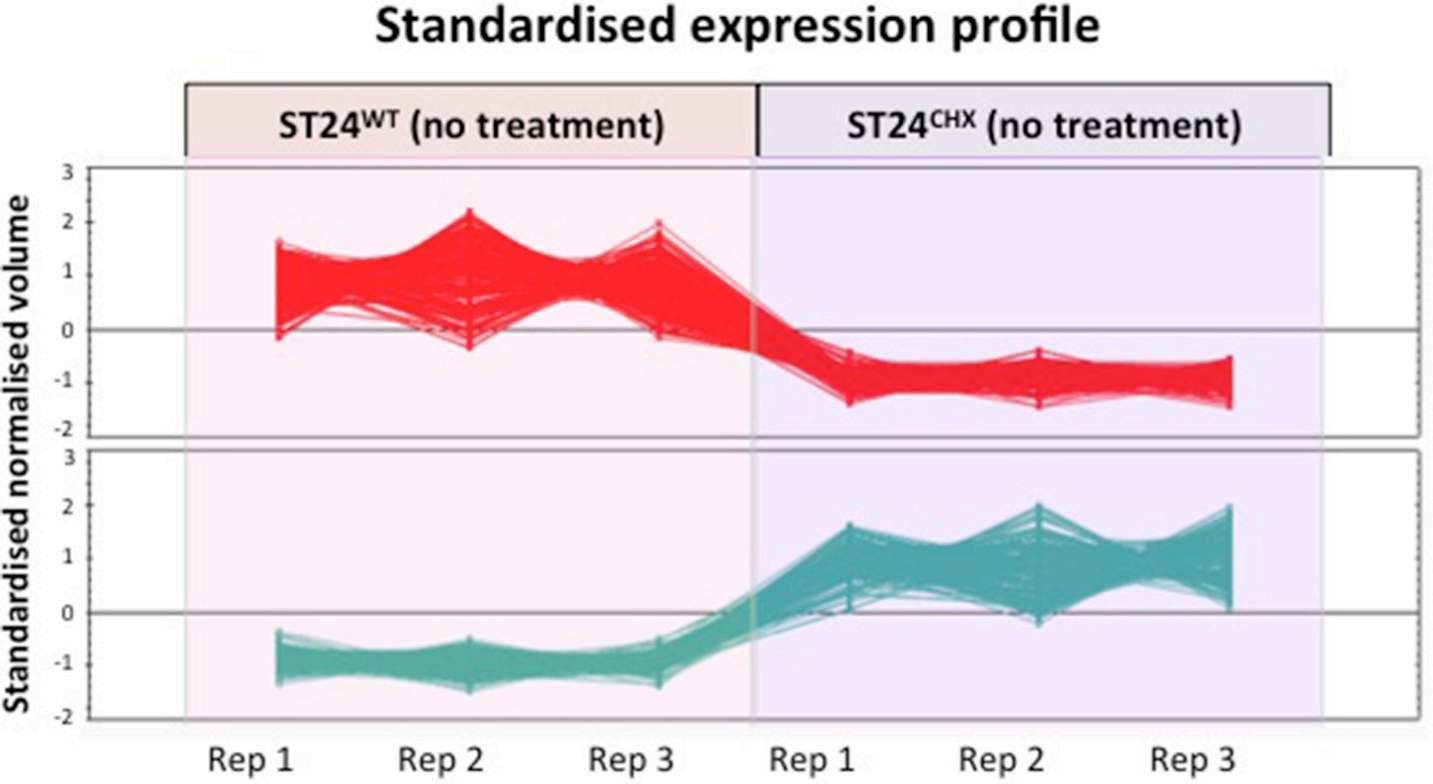
**Differential expression of proteins between the reference strain ST24^WT^ and mutant ST24^CHX^**. Profiles indicated in red represent down-regulated proteins in ST24^CHX^ relative to ST24^WT^, those in green are up-regulated. The data points represent the normalized expression level of individual protein spots (differentially expressed between the wild-type and the mutant), identified using Progenesis SameSpots software (^©^ Nonlinear Dynamics Ltd., Newcastle, UK). The various replicates of the same protein spot, between the reference and the mutant are linked with lines.

Proteomic analysis revealed a total of 470 protein spots differentially expressed, without chlorhexidine exposure, between ST24^WT^ and ST24^CHX^ (>2-fold change, *p* < 0.05). Among the differentially expressed proteins, 208 were up-regulated and 262 were down-regulated in ST24^CHX^ relative to the reference isolate (Figure 8). Of the up-regulated proteins a total of 48 protein spots were identified with statistical significance and these corresponded to 34 individual proteins (Table 2, Tables S4, S5). These were divided into three functional categories; general cell metabolism (24 proteins), stress response (5 proteins) and transport and permeability (5 proteins). Up-regulated proteins are indicated in green in Figure 8, denoted by the green traces in Figure 9, and functionally categorized in Table 2.

**Table 2:** **Identification and functional classification of proteins differentially expressed between the reference, chlorhexidine susceptible, *Salmonella* Typhimurium ST24^WT^, and the isogenic chlorhexidine tolerant mutant ST24^CHX^**.

**Spot #**	**Protein name**	**Function**	Functional category^*^	***p*-value**	**Fc^†^**	**GI number**
**PROTEINS UP-REGULATED**						
290	Anaerobic dimethyl sulfoxide reductase chain A (DmsA)	Terminal electron acceptor, reduction of DMSO to DMS	Anaerobic metabolism, Energy metabolism	4.12E-05	3.40	gi|323210416
202, 216	Bifunctional acetaldehyde-CoA/alcohol dehydrogenase (AdhE)	Reduction of acetyl-CoA to acetaldehyde and then ethanol	Anaerobic metabolism, Energy metabolism	1.48E-02	18.03	gi|16760134, gi|16760134
135, 327, 334, 344, 359	Formate acetyltransferase 1 (PflB)	Conversion of pyruvate and CoA to formate and acetyl-CoA	Anaerobic metabolism, Energy metabolism	7.21E-04, 2.83E-05, 6.39E-05, 5.26E-05, 3.10E-04	6.53, 10.05, 7.45, 10.43, 3.71	gi|213029313, gi|16759843
177	Succinate dehydrogenase flavoprotein subunit (SdhA)	Fumarate and succinate interconversion, under anaerobic conditions	Anaerobic metabolism, Energy metabolism	2.65E-05	8.43	gi|261245997
861, 869	Glyceraldehyde-3-phosphate dehydrogenase (GapA)	Conversion of glyceraldehydes-3-phosphate to D-glycerate 1,3-bisphosphate. Glycolysis	Energy metabolism	5.74E-05, 7.95E-11	9.90, 15.34	gi|16760605
554	Phosphoglyceromutase (PmgI)	Conversion of 2-phospho-D-glycerate to 3-phospho-D-glycerate. Glycolysis	Energy metabolism	5.18E-04	2.70	gi|213023081
279	Pyruvate kinase (PykF)	Conversion of phosphoenolpyruvate to pyruvate. Glycolysis	Energy metabolism	4.21E-05	5.72	gi|197362573
275, 349	Dihydrolipoamide acetyltransferase (E2) (AceF)	Component of the pyruvate dehydrogenase complex	Energy metabolism	2.03E-07, 9.76E-06	6.99, 9.91	gi|62178722
363, 368	Transketolase (TktA)	Transfer of a ketol group between several donors and acceptors, links glycolysis and the pentose phosphate pathway	Carbohydrate metabolism	1.53E-04, 2.11E-04	4.33, 4.61	gi|213850121, gi|301159632
936	Ribose-phosphate pyrophosphokinase (PrsA)	Conversion of D-ribose-5-phosphate to 5-phospho-alpha-D-ribose 1-diphosphate (PRPP). Pentose-phosphate pathway	Carbohydrate metabolism, Co-factor metabolism	1.11E-06	3.26	gi|16760673
1089	2-deoxyribose-5-phosphate aldolase (DeoC)	Conversion of 2-deoxy-D-ribose 5-phosphate to D-glyceraldehyde 3-phosphate and acetaldehyde. Pentose-phosphate pathway	Carbohydrate metabolism, Nucleotide metabolism	1.07E-04	4.22	gi|320089000
568	D-mannonate oxidoreductase (UxuB)	Conversion of D-fructuronate to D-mannonate. Pentose glucuronate interconversion	Carbohydrate metabolism	4.17E-06	4.06	gi|16761914
727	Mannitol-1-phosphate 5-dehydrogenase (MtlD)	Converts mannose-1-phosphate to fructose-6-phosphate	Carbohydrate metabolism	8.98E-05	3.65	gi|16766971
613	Alpha-galactosidase (MelA)	Hydrolysis of terminal, non-reducing alpha-D-galactose residues in alpha-D-galactosides; galactomannans, galactose oligosaccharides and galactolipids. Glycero/sphingolipid metabolism	Lipid metabolism, Carbohydrate metabolism, Anaerobic metabolism	2.89E-04	3.65	gi|16767548
585	Glycerol kinase (GlpK)	Conversion of glycerol to glycerol-3-phosphate, synthesis of triglycerides and glycerophospholipids	Lipid metabolism, Anaerobic metabolism	2.46E-06	9.03	gi|16762349
782	Galactonate dehydratase (DgoD)	Conversion of D-galactonate to 2-dehydro-3-deoxy-D-galactonate, glycero/sphingolipid metabolism	Carbohydrate metabolism	2.32E-05	4.03	gi|224585626
678	Serine hydroxymethyltransferase (GlyA)	Conversion of 5,10-methylenetetrahydrofolate and glycine to tetrahydrofolate and L-serine	Cofactor metabolism	3.92E-06	5.43	gi|213163620
1061	Pyridoxamine kinase (PdxY)	Conversion of pyridoxal (vitamin B6) to pyridoxal 5’-phosphate	Cofactor metabolism	3.43E-04	2.76	gi|16764798
1151	Dihydropteridine reductase (NfnB)	Conversion of 5,6,7,8-tetrahydropteridine to 6,7-dihydropteridine. Folate biosynthesis	Cofactor metabolism	1.28E-03	1.93	gi|238911529
1032	2-hydroxy-3-oxopropionate reductase (GarR)	Conversion of glycerate to 2-hydroxy-3-oxopropanoate, produces of NADH	Other	2.39E-03	2.43	gi|25283646
789	Glucose-1-phosphatase/inositol phosphatase (Agp)	Hydrolysis of phosphate from α-D-glucose-1-phosphate and 1-D-myo-inositol-hexakisphosphate. Glucose and inositol metabolism	Carbohydrate metabolism	5.84E-07	8.81	gi|323212400
37, 38, 240, 254, 262,	Elongation factor G (FusA)	Required for the catalysis of the GTP-dependent translocation step during translation elongation	Transcription, ribosomal structure and translation	1.54E-05, 2.80E-05, 1.11E-06, 2.38E-04, 9.20E-07,	6.45, 7.51, 9.05, 5.64, 9.26,	gi|16762837, gi|213586434
425	Prolyl-tRNA synthetase (ProS)	Member of the aminoacyl-tRNA synthetases	Transcription, ribosomal structure and translation	4.75E-05	4.79	gi|162139614
1127	Heat shock protein (GrpE)	Prevents aggregation of denatured proteins, in association with dnaK, dnaJ	Stress response	8.48E-05	2.28	gi|340000338
1115	DNA-binding transcriptional regulator (PhoP)	Cytoplasmic regulator of phoP/phoQ, controls the transcription of genes involved in virulence, transport and LPS modification	Stress response, regulation	8.28E-04	3.02	gi|16764586
732	Peptidyl-prolyl cis-trans isomerase (SurA)	Facilitate the proper folding of proteins in the periplasm	Stress response	1.34E-03	3.72	gi|16759087
238	Outermembrane protein assembly factor (YeaT)	Forms a complex with YfgL, YfiO, and NlpB. Involved in outer membrane protein biosynthesis and assembly	Stress response	2.57E-04	2.06	gi|16763614
418	Heat shock protein 90 (HtpG)	Molecular chaperone protein with ATPase activity	Stress response	2.85E-05	4.44	gi|161615315
1090	Osmolarity response regulator (OmpR)	Part of the EnvZ-OmpR system, involved in the regulation of numerous genes including the outer membrane porin genes in response to osmolarity	Transport/Permeability, regulation	8.48E-05	2.00	gi|15803909
962	Phosphate transporter ATP-binding protein (PstB)	Component of the phosphate starvation ABC transporter (pstABCS)	Transport/Permeability	3.44E-05	3.33	gi|16762475
1021	Amine ABC transporter, periplasmic amine-binding protein (YehZ)	Uptake protein for the transport of quaternary amines	Transport/ Permeability	1.11E-03	2.81	gi|323231218
902	Mannose-specific enzyme IIAB (ManX)	Mannose specific component of the phosphotransferase system	Transport/ Permeability, Carbohydrate metabolism	3.16E-04	3.01	gi|16765171
456	Putative ABC transporter, ATP-binding protein (YjjK)	Hydrolyses ATP, coupled to the translocation of a substrate across the membrane.	Transport/Permeability	3.99E-05	3.38	gi|168262364
**PROTEINS DOWN-REGULATED**						
606, 607	Pyruvate kinase (PykF)	Conversion of phosphoenolpyruvate to pyruvate. Pyruvate metabolism	Energy metabolism	6.86E-04, 2.85E-05	450, 534	gi|213855848, gi|161503526
906	Glyceraldehyde-3-phosphate dehydrogenase (GapA)	Conversion of glyceraldehydes-3-phosphate to D-glycerate 1,3-bisphosphate. Glycolysis	Energy metabolism	7.84E-05, 1.85E-04	11.45, 3.84	gi|16760605,
914	Phosphate acetyltransferase (Pta)	Conversion of acetyl-CoA and phosphate to CoA and acetyl phosphate. Glycolysis	Energy metabolism	1.21E-04	18.71	gi|322652083
965, 946	Phosphopyruvate hydratase (Eno)	Conversion of 2-phospho-D-glycerate to phosphoenolpyruvate. Glycolysis	Energy metabolism	1.65E-06, 3.16E-08	5.91, 16.11	gi|297521596
911	Phosphofructokinase (FruK)	Conversion of D-fructose-6-phosphate to D-fructose 1,6-bisphosphate	Energy metabolism	1.63E-04	4.75	gi|16761146
970, 1159	Phosphoglycerate kinase (Pgk)	Conversion of 3-phospho-D-glycerate to 3-phsopho-D-glyceroyl phosphate. Glycolysis	Energy metabolism	5.62E-06, 4.22E-08	17.35, 10.75	gi|224584862
1265	Acetate kinase (AckA)	Conversion of acetate to acetyl phosphate. Pyruvate metabolism	Energy metabolism	6.88E-05	44.55	gi|213581081
713	Fumarate reductase, flavoprotein subunit (FrdA)	Conversion of succinate to fumarate	Anaerobic metabolism, Energy metabolism	2.07E-06	9.62	gi|326626034
641	Bifunctional acetaldehyde CoA/alcohol dehydrogenase (AdhE)	Reduction of Acetyl-CoA to acetaldehyde and then to ethanol	Anaerobic metabolism, Energy metabolism	2.43E-04	4.40	gi|323270918
778	F_0_F_1_ ATP synthase beta subunit (AtpD)	Beta subunit of membrane-bound ATP synthase	Energy metabolism	5.11E-06	12.54	gi|112791348
1076	F_0_F_1_ ATP synthase subunit alpha (AtpA)	Alpha subunit of membrane-bound ATP synthase	Energy metabolism	2.42E-06	10.14	gi|213852796
1111, 1263	Uridine phosphorylase (Udp)	Conversion of uridine and phosphate to uracil and alpha-D-ribose 1-phosphate	Nucleotide metabolism	1.70E-06, 4.48E-05	6.40, 9.38	gi|158428692, gi|213421695
1191	Uracil phosphoribosyltransferase (Upp)	Conversion of UMP and diphosphate to uracil and 5-phospho-alpha-D-ribose 1-diphosphate	Nucleotide metabolism	5.56E-05	2.33	gi|213419931
731	Inosine 5^′^-monophosphate dehydrogenase (GuaB)	Conversion of inosine monophosphate to xanthosine 5^′^-phosphate	Nucleotide metabolism	1.98E-03	1.95	gi|16765831
794	Fructose 1, 6-bisphosphatase II (GlpX)	Conversion of D-fructose 1,6-bisphosphate to D-fructose 6-phosphate. Mannose/Fructose metabolism	Carbohydrate metabolism	3.23E-05	13.86	gi|16767351
929	Transaldolase B (TalB)	Conversion of sedoheptulose 7-phosphate and D-glyceraldehyde 3-phosphate to D-erythrose 4-phosphate and D-fructose 6-phosphate	Carbohydrate metabolism	2.46E-05	8.87	gi|16759000
604	Lysine decarboxylase (CadA)	Role in pH homeostasis. Conversion L-Lysine to cadaverine and carbon dioxide	Amino acid/peptide transport/metabolism	3.90E-04	6.23	gi|213053291
636, 1119	Aspartate ammonia-lyase (AspA)	Conversion of L-aspartate to fumarate and ammonia	Amino acid/ peptide transport/ metabolism	4.83E-04, 3.27E-08	7.71, 12.42	gi|213582259, gi|167553587
1121	Aminoacyl-histidine dipeptidase (PepD)	Metabolism of glutathione and amino acids	Amino acid/peptide transport/metabolism	1.11E-03	2.81	gi|16763698
1262	Enoyl-(acyl carrier protein) reductase (FabI)	An NADH-dependent trans-2-enoyl-ACP reductase. A key regulator of fatty acid biosynthesis	Lipid metabolism	5.25E-07	16.31	gi|16765044
1225,1323	Inorganic pyrophosphatase (Ppa)	Conversion of one molecule of pyrophosphate to two phosphate ions	Other	1.46E-05, 3.49E-06	1.61, 10.38	gi|16763234
113, 144	DNA directed RNA polymerase subunit beta (RpoB)	Component of DNA-dependent RNA polymerase- catalyses the transcription of DNA into RNA	Transcription, ribosomal structure and translation	5.49E-04, 4.81E-04	21.19, 10.59	gi|213161404
797	DNA-directed RNA polymerase subunit alpha (RpoA)	Component of DNA-dependent RNA polymerase- catalyses the transcription of DNA into RNA	Transcription, ribosomal structure and translation	9.66E-05	2.42	gi|293393287
957	Methionine aminopeptidase (Map)	Removes the N-terminal methionine from nascent proteins, release of N-terminal amino-acids	Transcription, ribosomal structure and translation	4.72E-05	2.19	gi|16759205
748	GTP-dependent nucleic acid-binding protein (YchF)	May act as a translation factor	Transcription, ribosomal structure and translation	5.47E-05	2.00	gi|16759205
705, 708, 918, 1166,	30S ribosomal protein S1 (RpsA)	Structural ribosomal protein	Transcription, ribosomal structure and translation	1.62E-08, 1.55E-07, 7.64E-05, 3.05E-05,	15.82, 42.48, 8.12, 17.03	gi|213418378, gi|213027002, gi|213021855
1321	30S ribosomal protein S2 (RpsB)	Structural ribosomal protein	Transcription, ribosomal structure and translation	2.79E-07	40.92	gi|16759206
488	Translation initiation factor IF-2 (InfB)	Promotes the GTP-dependent binding of the initiator tRNA to the small subunit of the ribosome	Transcription, ribosomal structure and translation	8.57E-06	11.34	gi|213423445
1149, 1336	Elongation factor Ts (Tsf)	Catalyses the release of guanosine diphosphate from EF-Tu	Transcription, ribosomal structure and translation	1.69E-07, 6.07E-06	13.38, 25.60	gi|213028021, gi|213618628
265, 353, 386, 387	Elongation factor G (FusA)	Catalyses the translocation of the tRNA and mRNA down the ribosome at the end of each round of polypeptide elongation	Transcription, ribosomal structure and translation	1.70E-04, 1.48E-04, 1.42E-05, 6.48E-05	6.59, 8.14, 6.37, 4.12	gi|16762837, gi|213416464
1028, 1064	Elongation protein Tu (TufB)	Mediates the entry of aminoacyl-tRNA into a free site on the ribosome	Transcription, ribosomal structure and translation	1.96E-03, 5.49E-06	19.62, 7.71	gi|315253052
1082	Putative elongation factor (YeiP)	Facilitates translation	Transcription, ribosomal structure and translation	2.78E-07	16.87	gi|56127134
887	Lysyl-tRNA synthetase (LysS)	Member of the aminoacyl-tRNA synthetases	Transcription, ribosomal structure and translation	1.53E-04	6.25	gi|16766341
453	Molecular chaperone (DnaK)	Involved in DNA replication and in response to hyperosmotic shock	Stress response	9.19E-06	8.07	gi|161504855
440, 1005	High temperature protein G (HtpG)	Molecular chaperone protein with ATPase activity	Stress response	5.56E-05, 2.67E-04	21.70, 75.16	gi|320084762, gi|213022746
1074	ATP dependent protease binding subunit (ClpB)	Component of Clp protease	Stress response	7.20E-04	7.17	gi|4102206
728	ATP-dependent protease ATP-binding subunit (HslU)	ATPase subunit of a protease degradation complex, ATP binding and hydrolysis component	Stress response	1.33E-08	10.93	gi|213051554
753, 757, 1247	Phosphoenolpyruvate-protein phosphotransferase (PtsI)	Component of the PTS system. Transport and conversion of phosphoenolpyruvate to pyruvate	Transport/Permeability, energy metabolism	1.73E-07, 1.16E-05, 6.10E-07	39.30, 29.51, 20.87	gi|213051554, gi|213586857
947	Flagellar hook-associated protein (FlgL)	Role in flagellar biosynthesis, hook associated protein	Flagella and chemotaxis	3.48E-04	5.31	gi|56413829

* Functional categories assigned according to KEGG.

† *Fc, fold change. (see also Figure 8)*.

Of the down-regulated proteins a total of 57 were identified with statistical significance and these corresponded to 39 proteins (Table 2, Tables S4, S5). The latter could be divided into four functional categories including; general cell metabolism (33 proteins), stress response (4 proteins), motility (1 protein) and transport and permeability (1 protein). Down-regulated proteins are indicated in red in Figure 8, denoted by red traces in Figure 9 and categorized, as before in Table 2.

**Figure 10:**
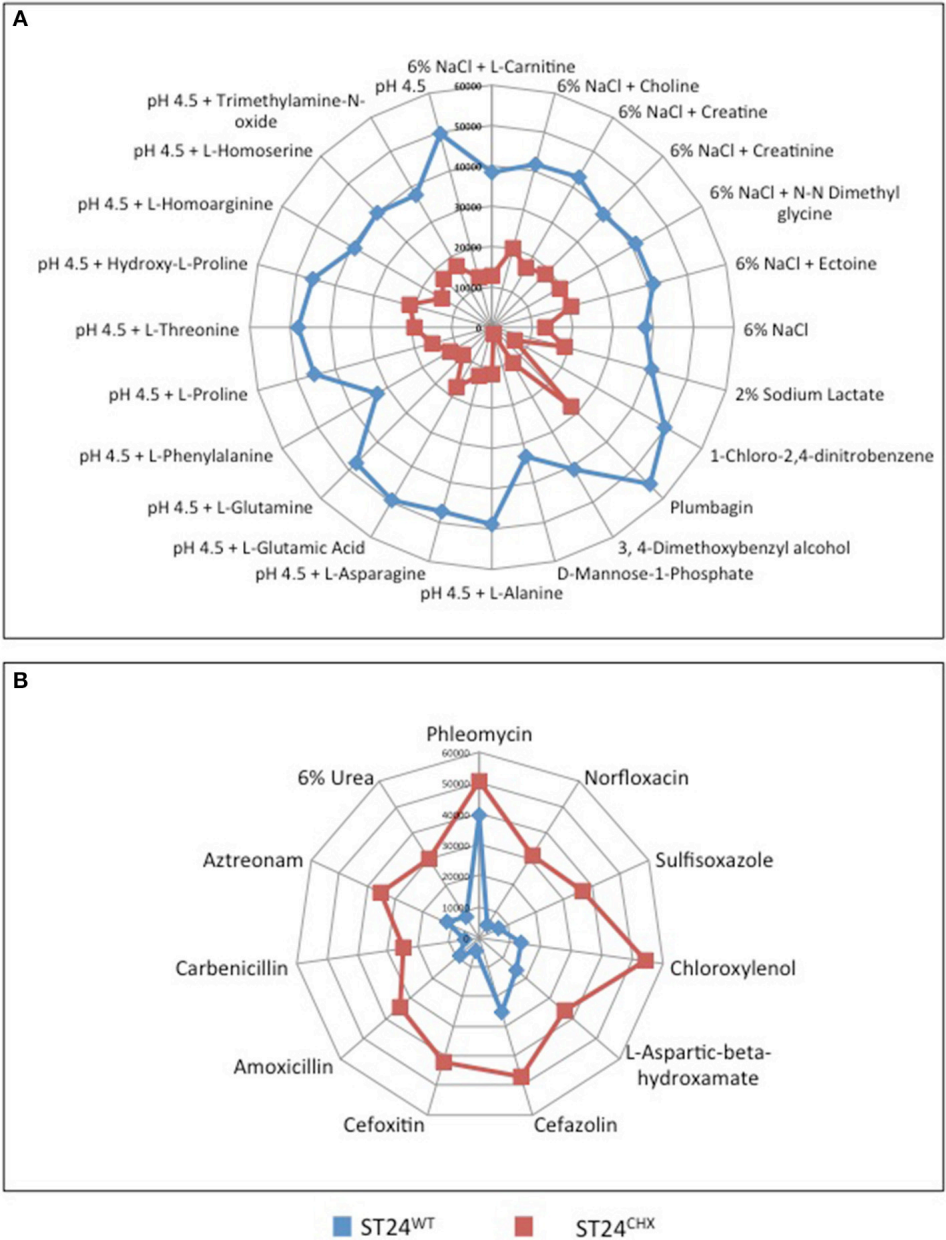
**Metabolic differences that distinguish the sensitive reference ST24^WT^, from the chlorhexidine tolerant mutant ST24^CHX^ on (A) osmolytes where enhanced respiration was recorded in the reference strain and (B) substrates where enhanced respiration was recorded in the mutant isolate**. The blue lines represent the metabolism of ST24^WT^ in Omnilog units, whilst the red lines represent the metabolism of ST24^CHX^. The center of the graph (0 Omnilog units) equates to no metabolic activity. The periphery of the graph (60,000 Omnilog units) equates to maximum metabolic activity. Each spoke of the graph represents a different compound with the box on each spoke indicating the metabolic activity of ST24^WT^/ST24^CHX^ in the presence of that particular compound.

Phenotypes expressed by ST24^WT^ and ST24^CHX^ were compared, using the Omnilog™ phenotypic microarray platform. No significant differences in the growth rates of ST24^WT^ and ST24^CHX^ were detected in MH broth or in the Omnilog™ inoculation fluid (data not shown). Data from the phenotypic microarray showed, however, that alterations in the respiration of the chlorhexidine tolerant ST24^CHX^, relative to susceptible ST24^WT^, were apparent when grown on a variety of substrates and in the presence of antimicrobial compounds. All of these are listed in Table S6 and displayed as a series of heatmaps in Figures S1A and B. An increased respiration rate was also noted for ST24^CHX^, indicating enhanced tolerance, to 5 cell wall damaging agents, 2 DNA damaging agents, a folate synthesis inhibitor, and a glutamate up-take inhibitor (Figure 10). Conversely, ST24^CHX^ was found to be more susceptible to 19 osmolytes (Figure 10), 16 cell wall/cell membrane damaging agents, 9 protein synthesis inhibitors, 6 respiration un-couplers, 3 oxidizing agents, and 11 agents causing damage to DNA or inhibiting DNA synthesis, replication or transcription, when compared to ST24^WT^.

### Modifications in general cell metabolism comparing ST24^WT^ and ST24^CHX^

Relative to ST24^WT^ the chlorhexidine tolerant mutant ST24^CHX^ displayed multiple alterations in the general cell metabolism, at both the transcriptomic and proteomic levels (Encheva et al., 2009). The alterations were independent of chlorhexidine exposure (detected in ST24^CHX^ with and without exposure to the same agent). These metabolic modifications were similar to those detected in ST24^WT^ following chlorhexidine exposure, described above. However, metabolic changes in ST24^CHX^ were more wide ranging (Figure 5B).

An increase was detected in ST24^CHX^ in the level of pyruvate formate lyase. This enzyme catalyzes the non-oxidative fermentation of pyruvate to formate and acetyl-CoA, thus providing the main substrate for mixed acid fermentations. Additional up-regulation in multiple enzymes involved in mixed acid fermentation was observed in the mutant isolate including an alcohol/aldehyde dehydrogenase enzyme, which converts acetyl-coA to acetaldehyde and then subsequently to ethanol. Additionally, there was a mutation in a putative formate transporter (Table 1). Furthermore, the anaerobic dimethyl sulfoxide (DMSO) reductase (*dmsA*) was up-regulated, an enzyme, which works as part of an electron transport chain along with, formate dehydrogenase. An increase was also noted in the expression of succinate dehydrogenase, which catalyzes the conversion of fumarate to succinate under anaerobic conditions. The genomic comparison identified a non-synonymous polymorphism in the α-subunit of oxaloacetate decarboxylase, an enzyme involved in the anaerobic citrate fermentation, a pathway that converts citrate to acetate, formate and CO_2_ (Bott, 1997). Multiple genes associated with propanediol utilization were also up-regulated in the mutant isolate, with and without chlorhexidine treatment (Table S1).

Given these observations, we hypothesize that chlorhexidine targets component/(s) of the respiratory chain. This phenotype could be expected to result in a reduction in oxygen consumption concomitant with a decrease in the proton motive force (PMF). This scenario would in turn result in reduced ATP production, which could be expected to have a pleiotropic effect leading, for example, to a reduction in nucleic acid and protein synthesis and ultimately the disruption of the structural integrity of the bacterial membrane. However, these detrimental effects may be circumvented should the bacterial cell divert the electron flow through the reduction of organic acids as described above. A similar situation has been previously reported where the oxidation of formate, catalyzed by formate dehydrogenase and succinate, by succinate dehydrogenase, afforded *E. coli* and *Salmonella* some protection from a cationic-based antimicrobial peptide (Barker et al., 2000). More recently, Cheung et al. reported that following chlorhexidine exposure, two enzymes involved in mixed acid fermentation, succinate dehydrogenase and lactate dehydrogenase, were both up-regulated in *E. coli* (Cheung et al., 2012), a feature that would appear to support our hypothesis. Nde et al. similarly found that chlorhexidine exposure in *P. aeruginosa* resulted in the down-regulation of several enzymes involved in oxidative phosphorylation and the up-regulation of enzymes involved in anaerobic metabolism, suggesting a dynamic shift in metabolic activity toward an anoxic profile. Nde and colleagues concluded that chlorhexidine suppressed energy metabolism *via* aerobic respiration.

In support of this hypothesis, an increase was observed in the expression of pyruvate dehydrogenase (quinone) at the transcriptional level in the chlorhexidine tolerant ST24^CHX^, with and without chlorhexidine treatment, relative to ST24^WT^. This enzyme catalyzes the conversion of pyruvate to acetate and, importantly, is coupled to the electron transport chain at the level of ubiquinone, in the terminal electron transport system. An increase in activity was similarly detected for the NADH dehydrogenase complex I, an enzyme involved in the anaerobic metabolism of fumarate. Several enzymes linked with glycerolipid metabolism, including the β-subunit of the anaerobic glycerol-3-phosphate dehydrogenase described above, were up-regulated in ST24^CHX^. This pathway shuttles electrons to the terminal electron transport chain at the level of succinate dehydrogenase. Enzymes in this pathway were up-regulated at both the transcriptomic and proteomic levels in ST24^CHX^ (Figures S2A and B). These alterations in electron flow in ST24^CHX^ were also reflected at the level of its metabolism when analyzed by the phenotypic microarray; wherein the mutant displayed an increased susceptibility to 6 respiration un-couplers (Figure S1 and Table S6).

Consistent with altered metabolism, the differential expression of genes involved in the utilization of other carbon sources were noted in ST24^CHX^ relative to ST24^WT^, independent of chlorhexidine exposure (Table 2, Table S1). At both the RNA and protein levels, several transporters for different sugars, along with enzymes for their conversion to intermediates of the glycolytic pathway, were up-regulated in the mutant, including those for mannose, mannitol, 1-D-myo-inositol-4-phosphate, sorbitol and galactose (Table 2, Table S1). Carbon and electron flow within glycolysis and pyruvate metabolism was also altered with an increase in some enzymes and a decrease in others (Table 2, Table S1 and Figure S3). Interestingly, there was a marked increase in transketolase expression together with other enzymes of the pentose phosphate pathway (PPP) and pentose-glucuronate interconversions in ST24^CHX^. However, a decrease in transaldolase expression was noted (Table 2, Table S1). This feature suggested that the flow of carbon reducing power was away from glycolysis, being shuttled instead toward the PPP. Within this pathway, enzymes for the synthesis of the cofactor phosphoribosyl pyrophosphate (PRPP) were up-regulated (Table 2, Table S1 and Figure S4).

Based on the transcriptomic and proteomic data, several global regulators that were up-regulated in the mutant relative to ST24^WT^ may account for at least some of the transcriptomic, proteomic and phenotypic changes observed. As an example, up-regulation of *creB* was noted in ST24^CHX^. CreB is thought to be a global regulator (Avison et al., 2001), and its expression is induced by the fermentation of carbon sources through glycolysis and anaerobic reactions, similar to those described above (Cariss et al., 2008). It has been demonstrated that CreB can control the expression of *talA*, an enzyme associated with the shuttling of glyceraldehyde-3-phophate away from glycolysis to the non-oxidative PPP (Avison et al., 2001). This is consistent with the up-regulation of enzymes associated with the PPP pathway noted in our study. Furthermore, CreB is also known to positively regulate phosphate acetyltransferase (*pta*), which was also up-regulated in ST24^CHX^ at the transcriptomic and proteomic levels, along with acetate kinase (*ackA*), which was up-regulated at the protein level. Both of these enzymes are required for efficient carbon flux to support growth using sugars transported *via* the phosphotransferase system (PTS) (Avison et al., 2001), many of which were up-regulated in ST24^CHX^ (Table 2).

The ferric up-take regulator (*fur*) was up-regulated in the mutant, compared with its wild-type progenitor. Fur is involved in metal-dependent transcriptional regulation in response to different metal concentrations and the redox state of the bacterial cell (Escolar et al., 1999; Troxell et al., 2011). Several cellular targets known to be positively regulated by Fur displayed an increased expression in ST24^CHX^, including those involved in anaerobic metabolism, such as dimethyl sulfoxide reductase (*dmsA*), which was up-regulated at both the RNA and protein levels, and those involved in glycerol/glycerolipid metabolism (*glpAB*), which were up-regulated at the RNA level. Decreased expression of cellular targets known to the negatively regulated by Fur, including those involved in glycolysis was also noted; for example, phosphofructokinase (*pfkA*), which was detected as down-regulated at the RNA and protein levels. It is reasonable to hypothesize that both Fur and CreB are important regulators of the chlorhexidine tolerant phenotype described in this study. Current work involving knock-out studies is on-going in order to further elucidate the possible role of these regulators in the chlorhexidine tolerance response.

### Modifications in membrane structural and transport systems between ST24^WT^ and ST24^CHX^

Relative to ST24^WT^ the chlorhexidine tolerant mutant ST24^CHX^ displayed multiple alterations in peptidoglycan synthesis/cross-linking, independent of chlorhexidine exposure. These alterations were similar to those detected in ST24^WT^ following a 30 min sub-lethal chlorhexidine exposure, described earlier. However, modifications were more extensive in ST24^CHX^, with additional markers being detected. These included the up-regulation of the peptidoglycan synthetic gene *murF* and those involved in cross-linking; *ppbC, pbpG*. A reduction in expression was noted for loci encoding component parts of the LPS structure, such as *rpfU*, *rfbF*, and *rfbH*. Conversely, increased expression was observed in 2 enzymes associated with LPS biosynthesis, namely *kdsA*, which plays a key role in LPS biosynthesis and *lpxK* which catalyses the transfer of a phosphate from ATP to the 4′-position of the lipid A disaccharide (Table S1). A SNP was detected in the *lptC*-encoding gene, a component of a LPS export system (Tran et al., 2010). A mutation in this gene was previously found to impart a reduced susceptibility to the bile salt sodium deoxycholate in *Salmonella* (Hernández et al., 2012).

Based on the data obtained in this study, extensive modifications in the expression of genes/proteins associated with permeability were noted, suggesting modifications to the membrane function as important determinants of tolerance. These membrane alterations were similarly reflected in the data obtained from the phenotypic microarray. Some of the modifications noted in the membrane/cell wall structure of the mutant correlated with an increased sensitivity to 20 osmolytes (Figure 10A, Figure S1A and Table S6B). Similarly, the mutant exhibited an increased susceptibility to cell wall disrupting agents, including vancomycin and three β-lactam antimicrobials, along with 12 other membrane active agents, including colistin, a receptor for which was up-regulated (Figure S1B and Table S6B). Conversely, modifications in the cell wall also correlated with an increased tolerance to 5 clinically important, cell wall damaging agents, including 2 cephalosporins and 2 β-lactams (Figure 10B, Figure S1B and Table S6A). Earlier data reported the susceptible profile of ST24^WT^ to b-lactam and cephalosporin antibiotics (Condell et al., 2012a). Based on these observations, it is tempting to speculate that the nature of the diverse phenotype reported here (Figure 10B and Tables S6A,B) may be reflective of changes occurring at the level of the bacterial membrane, in response to the stress imposed following exposure to chlorhexidine.

### Efflux pump activity comparisons between ST24^WT^ and ST24^CHX^

Similar efflux expression patterns were noted in ST24^CHX^ and ST24^WT^, as described above (Table S1). In this case, a reduction in the expression of *acrB* and *acrF*, components of the AcrAB-TolC and AcrEF-TolC tripartite efflux systems (Zheng et al., 2009) was recorded. Interestingly, a mutation in *ramR* was also identified in ST24^CHX^ (Table 1). RamR acts as a repressor of *ramA* (O'Regan et al., 2009), a known global transcriptional regulator of these latter efflux systems (Zheng et al., 2009). Mutations in *ramR* have previously been documented and these are associated with a de-repression of *ramA* and consequently the up-regulation of the aforementioned efflux systems (Abouzeed et al., 2008; O'Regan et al., 2009). However, in this case, it is possible that a mutation in *ramR* noted in ST24^CHX^, and which occurred within a *helix-turn-helix* motif, resulted in a reduction in *ramA* expression and, as a consequence, lowered the expression of both efflux systems. Moreover, in ST24^CHX^ up-regulation in the expression of *emrR*, a negative regulator of the efflux system *emrAB-tolC* (Tanabe et al., 2009) was observed (Table S1) and this may lead to a more pronounced repression of this system. Similarly, there was also a decrease in the expression of the multi-drug efflux associated gene *ydhE*.

In respect of other efflux systems in ST24^CHX^, members of the major facilitator superfamily (MFS family) including *bcr* and *smvA* (Paulsen and Brown, 1996) and the ABC superfamily multi-drug transporter family; *mdlB* (Sulavik et al., 2001) were up-regulated at the transcriptional level (Table S1). Precisely how efflux systems might play a role in the susceptibility of bacteria toward chlorhexidine remains to be elucidated.

### SOS response comparisons between ST24^WT^ and ST24^CHX^

Relative to ST24^WT^ the chlorhexidine tolerant mutant ST24^CHX^ displayed multiple alterations in the SOS response, independent of chlorhexidine exposure. Adaptations noted were similar to those detected in ST24^WT^ following chlorhexidine exposure. These modifications were more extensive in ST24^CHX^. An increased expression of 12 genes associated with DNA damage repair was observed in ST24^CHX^, relative to ST24^WT^ (Table S1). The up-regulated genes included those associated with the SOS response-DNA damage repair system, consisting of *recR*, *recO*, *recG*, *uvrA*, *uvrD*, and *ruvB* (Janion, 2008), indicating chlorhexidine tolerance was associated with an induction of this repair system. This alteration in DNA metabolism in the mutant was further supported by data from the phenotypic microarray. Chlorhexidine tolerance correlated with a decrease in susceptibility to 2 DNA damaging agents (Figure S1B and Table S6). The hypothesis that an induction of the DNA repair systems along with DNA binding proteins might represent the bacterial response to mitigate the DNA cross-linking effects of chlorhexidine appears to hold with regards ST24^CHX^. However, the specific DNA defense system induced in response to chlorhexidine correlated with a reduced tolerance to other DNA damaging agents; an increase in susceptibility to 10 such agents was detected from the phenotypic microarray (Figure S1B and Table S6).

### A comparison of transcription and translation between ST24^WT^ and ST24^CHX^

As was discussed above, ST24^WT^ in response to chlorhexidine exposure showed a decrease in the expression of transcription and translation associated genes and proteins. Similarly a decrease in the expression of genes associated with these cellular functions was noted in ST24^CHX^, relative to ST24^WT^, in this case the decrease was independent of chlorhexidine exposure (Table 2, Table S1). Reduced expression of *rpoA* at the RNA and protein levels were recorded, along with a reduction in the expression of the β-encoding sub-unit (*rpoB*) at the protein level. Furthermore, some 17 ribosomal structural genes were down-regulated, and 2 of these were also affected at the protein level. The ribosomal maturation factor (encoded by *yfiA*) and several translation factors including those for translation initiation and elongation were down-regulated (Table 2, Table S1). Two SNPs were detected in the mutant at 2 loci coding for ribosomal RNA (rRNA); 1 coding for a 23S rRNA and the other for a 16S rRNA. There were also 2 SNPs detected at 35 and 37 bp up-stream of a 16S rRNA, and these polymorphisms may have an effect on transcription (Table 1).

Mutations associated with changes in the expression of components of the cellular transcription and translation machinery, were reflected in the metabolism recorded from the phenotypic microarray data. ST24^CHX^ displayed an increased tolerance to an agent affecting protein synthesis, namely L-aspartic-beta-hydroxamate and an increased sensitivity to rifampicin an inhibitor of RNA polymerase, along with seven inhibitors of protein synthesis, including drugs of the macrolide, polyketide and aminoglycoside classes (Figure S1B and Table S6). These results further support the hypothesis that chlorhexidine affects bacterial protein synthesis as a component of its mechanism of action, as previously described by Nde et al. (2009).

### Comparison of the virulence features of ST24^WT^ and ST24^CHX^

ST24^WT^ following exposure to chlorhexidine exhibited a decrease in the expression of virulence associated genes. Similarly a decrease in the expression of virulence genes was noted in ST24^CHX^, relative to ST24^WT^, once more in this case the decrease was independent of chlorhexidine exposure. A down-regulation was noted in 12% of the virulence genes listed from KEGG, comprising SPI-1 through -5 in ST24^CHX^ relative to ST24^WT^ (Figures 2, 7). Two mutations in *skiK1*, a gene coding for a SPI-6 associated component of a type VI secretory system, were detected in the mutant isolate, along with a mutation in a second SPI-6 gene; coding for a protein of the Rhs family, and a mutation in a SPI-1 associated regulator protein (*avrA*). These results further indicate a relationship between chlorhexidine and a reduction in virulence gene expression. Further work confirmed a significantly attenuated virulence potential in ST24^CHX^, and a decrease in survival in THP-1 macrophages compared with ST24^WT^ was also noted (data not shown). Previous work demonstrated a relationship between central metabolism, the TCA cycle and the intracellular survival of *S*. Typhimurium (Bowden et al., 2010). It is possible that the reduced survival of ST24^CHX^ is linked with the metabolic modifications it displayed.

### Altered phosphate metabolism in ST24^CHX^

Changes in phosphate metabolism were detected in ST24^CHX^, but not in ST24^WT^ after sub-lethal chlorhexidine exposure. Inorganic phosphate is an essential element required for the phosphorylation of nucleic acids, lipids, sugars, proteins and for cell signaling. *Salmonella* species possess two major systems which function in the up-take of inorganic phosphate; the PTS and PIT systems. Both exhibited altered levels of expression in ST24^CHX^ relative to the reference isolate. The *ptsABCS* system codes for a phosphate starvation ABC transporter that is induced under conditions where limited phosphate exists. In the mutant, this transporter was up-regulated based on the protein data (Table 2). Additionally, a SNP was detected in the PIT system in ST24^CHX^ which introduced a premature stop codon (T*G*G-T*A*G) in the distal part of *pitA* (Table 1). PitA is a low affinity transporter thought to be constitutively expressed in *Salmonella* (Jackson et al., 2008). These features may indicate an increase in phosphate up-take in the mutant isolate. A mutation was also noted in a phosphate-linked antiporter (*pgtP*), which functions in the low affinity exchange of cytoplasmic inorganic phosphate for external phosphoglycerate (Varadhachary and Maloney, 1991). A down-regulation of two genes known to induce the expression of the latter antiporter, *pgtC* and *pgtB*, was also previously reported (Yang et al., 1988). The abundance of the inorganic pyrophosphatase enzyme (encoded by the *ppa* gene) was reduced in the mutant isolate (Table 2). These features suggest that an extensive change in phosphate metabolism had occurred in the mutant. Chlorhexidine is known to associate strongly with phosphate ions contained within the LPS (Tattawasart et al., 2000), and this biocide can form cytoplasmic complexes with phosphate containing moieties, which may lead to the precipitation of cytoplasmic contents (Cheung et al., 2012). Alterations in phosphate transport and its subsequent metabolism in the mutant appear to reflect these biocide-induced modifications following *in vitro* selection, wherein the cell may be acting to limit chlorhexidine adsorption to phosphate groups at the bacterial outer surface and consequently reduce the susceptibility of the bacterium to the biocide.

### Altered flagella gene regulation in ST24^CHX^

Modifications in flagella-mediated motility are an important factor in environmental adaptation and in adherence to various surfaces. Up-regulation of a number of flagella structural genes were detected in the ST24^CHX^ alone, independent of exposure to chlorhexidine, relative to ST24^WT^. These included flagellar hook-base body genes; *flgA*, *flgC*, *flgF*, *flgG*, *flgH*, *flgI, fliF, fliH, fill, fliM, fliO* (Table S1). The regulatory modification resulting in this up-regulation may be related to two SNPs detected in ST24^CHX^ one of which was located in the SL1344_4465 gene, a hypothetical transcriptional regulator of the NtrC family that may activate RpoN (σ^54^) (Table 1). σ^54^ in turn acts as a positive regulator of σ^28^ (*fliA*) (Dong et al., 2011). The action of σ^28^ is counteracted by an anti-σ^28^ sigma-factor, FlgM, which was down-regulated in the ST24^CHX^ mutant. This decreased expression may result in the overexpression of σ^28^, a phenomenon previously shown to result in increased expression of all flagellar genes of the temporally regulated *middle* gene promoters (Chilcott and Hughes, 2000). Based on our transcriptomic data, the genes identified that were up-regulated were all flagellar-middle genes. An increased swarm motility phenotype was also observed in the ST24^CHX^, compared to ST24^WT^, in the presence of low levels of chlorhexidine (data not shown). An up-regulation in the expression of the flagellar apparatus has previously been associated with a tolerance to antimicrobial agents in *Salmonella* (Kim et al., 2003; Kim and Surette, 2003; Butler et al., 2010) and may now be extended to chlorhexidine tolerance.

### Metabolic defense network elicited by exposure of *Salmonella* to chlorhexidine

**Figure 11:**
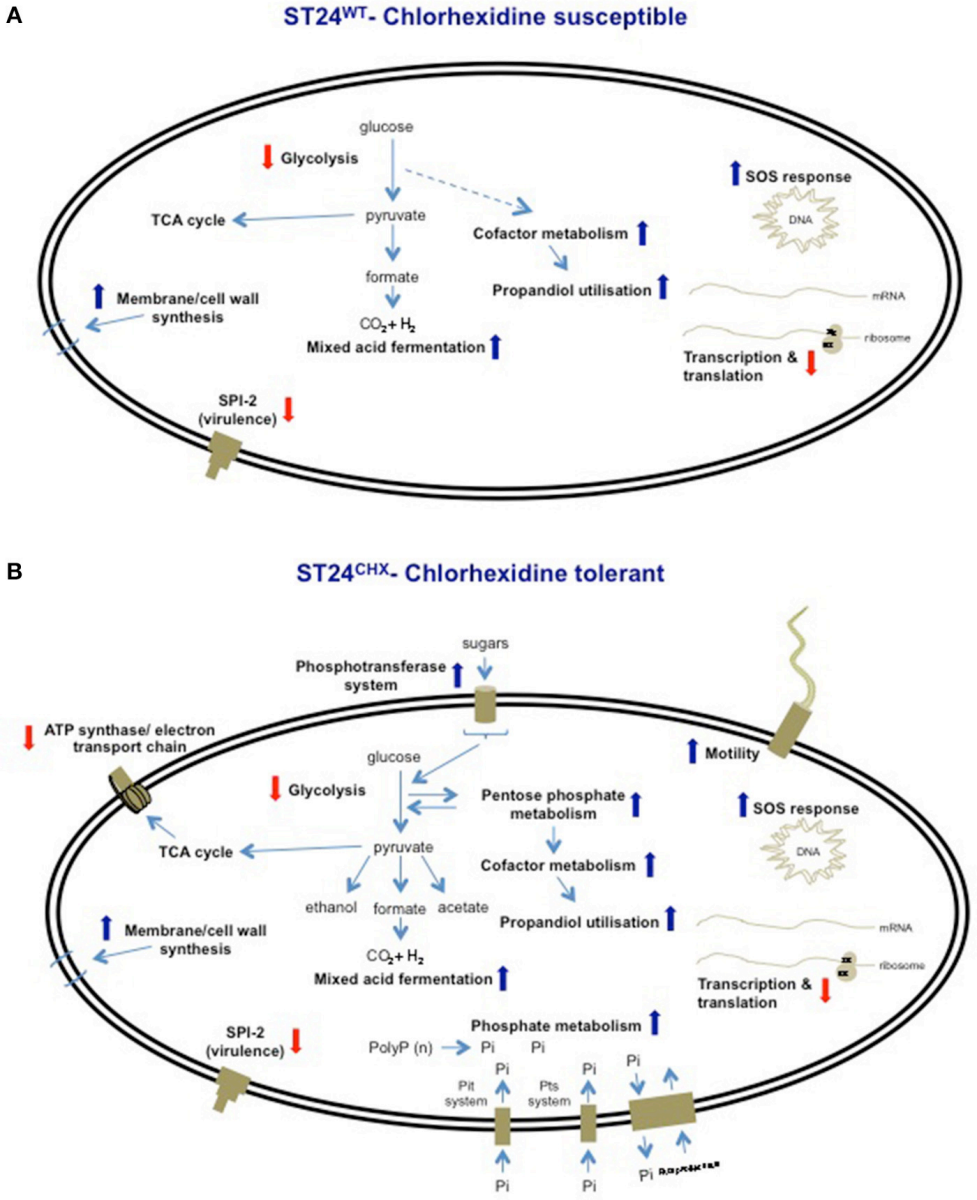
**Overview of cellular process altered by chlorhexidine, relative to the chlorhexidine-sensitive ST24^WT^, in (A) the same isolate following chlorhexidine exposure and (B) in the chlorhexidine tolerant ST24^CHX^, with and without chlorhexidine exposure**. Red arrowheads shown signify reduced expression and blue arrowheads signify increased expression.

Based on the observations outlined above describing alterations in expression of genes and proteins involved in general cell metabolism, membrane structure, efflux, the SOS-response, transcription and translation and virulence, it may be reasonable to hypothesize that exposure of ST24^WT^ to chlorhexidine elicits a distinct cellular response. Furthermore, these data point to the fact that chlorhexidine tolerance in ST24^CHX^ was also associated with broad cellular alterations; the mutant isolate demonstrated changes in general cell metabolism, membrane structure, efflux, the SOS-response, transcription and translation, virulence, phosphate metabolism and motility. Overlaps were noted between the alterations seen in ST24^WT^ following chlorhexidine exposure and changes in the geno-/phenotype in ST24^CHX^ relative to ST24^WT^, independent of chlorhexidine exposure. These features were suggestive of a general chlorhexidine defense network (Figure 11). Based on the transcriptomic profiling a common response was observed for 165 genes; wherein 51 were up-regulated and 114 genes were down-regulated in both isolates (Figure 2). Furthermore, the distribution of chlorhexidine-regulated genes between the functional categories was similar for both (Figure 3). The proposed network involved multiple components, which produced changes in the activity of several metabolic pathways, shifting toward an anoxic profile, a feature designed to mitigate the oxygen inhibitory effects of chlorhexidine. Moreover a common up-regulation in the SOS-response was observed and this may be part of the bacterial response to the DNA damaging effects of this agent. Similar modifications in protein systhesis and bacterial cell wall synthesis were observed, all designed to reduce the antimicrobial effects of chlorhexidine. Finally a decrease in the expression of virulence associated cell targets along with those associated with transcription and translation was detected in both isolates (Figure 11).

Furthermore, data from this study showed that the increased chlorhexidine tolerance of ST24^CHX^ was associated with more extensive alterations of the same components involved in this proposed defense network, when all of the functional categories were considered; relative to ST24^WT^(Figure 11). Interestingly, ST24^CHX^ also elaborated some unique responses including modifications in phosphate metabolism and alterations in the expression of chemotaxis and mobility associated genes that may contribute to the increased chlorhexidine tolerance of the mutant.

## Conclusions

This study investigated the bacterial response to chlorhexidine exposure at a number of biochemical levels in the bacterial cell. Results show that chlorhexidine elicits a broad range of effects on *Salmonella*, with an impact on central cellular processes including aerobic energy production and protein synthesis. This chlorhexidine defense network was conserved between the isogenic bacteria studied (Figure 11). Although ST24^CHX^ elaborated additional modifications, which extended the proposed defense network, a divergent defense response involving the up-regulation of additional targets was noted which may be mediated by CreB and Fur (Figure 11).

Results from this study when compared to those of Condell et al. (2012b) suggest that the number of cellular alterations and the ease at which a tolerant phenotype to an active biocide develops can differ hugely depending on the mechanism of action of the agent(s). A tolerant phenotype to the biocidal agent triclosan developed at a faster rate compared with the emergence of chlorhexidine tolerance (Condell et al., 2012a). The numbers of alterations in the proteome of the triclosan tolerant isolate, compared with its susceptible progenitor were fewer, at 33, compared to the 470 proteins differentially expressed between ST24^WT^ and ST24^CHX^. These differences in the number of alterations may reflect the mechanism of action of the two agents; triclosan has a cellular target, FabI, whereas chlorhexidine appears to have a broad spectrum mechanism.

Investigations of this nature are important in an effort to extend our understanding of how biocides act to eliminate bacteria, and the mechanisms by which a tolerance could develop. Such data are often lacking, even for the most commonly used biocides, and this information could assist the future refining and optimization of active formulations, to overcome potential tolerance mechanisms thereby improving biosecurity measures.

### Conflict of interest statement

The authors declare that the research was conducted in the absence of any commercial or financial relationships that could be construed as a potential conflict of interest.

## Supplementary material

Figure S1 **(A)** Comparative respiration of ST24^WT^, and the chlorhexidine tolerant mutant, ST24^CHX^, on PM plates 1–10, containing energy sources and osmolytes. Substrates where a significant difference in the respiration between ST24^WT^ and ST24^CHX^ was recorded are indicated with arrows and details are provided in Table S6. **(B)** Comparative respiration of ST24^WT^ and the chlorhexidine tolerant mutant, ST24^CHX^, on PM plates 11–20, containing antimicrobial compounds. Compounds where a significant difference in the respiration between ST24^WT^ and ST24^CHX^ were recorded, indicating a possible alteration in susceptibility, are indicated with arrows and details are provided in Table S6.

Figure S2 **Relative expression of enzymes that were determined to be differentially expressed and associated with (A) glycerolipid metabolism in the reference isolate ST24^WT^. (B)** Glycerolipid metabolism in the mutant ST24^CHX^, **(C)** amino acid metabolism in the reference isolate ST24^WT^, and **(D)** amino acid metabolism in the mutant ST24^CHX^. Enzyme names, descriptions, gene identifiers and fold-change values are given in Table 2, Tables S1, S4. Color blocks without a white diagonal line represent gene fold change values (Table S1), color blocks containing a white diagonal line represent protein fold change values (Table 2, Table S4).

Figure S3 **Relative expression of enzymes determined as differentially expressed and associated with carbohydrate metabolism in (A) the reference isolate ST24^WT^ and (B) the isogenic mutant ST24^CHX^**. Enzyme names, descriptions, gene identifiers and fold-change values are given in Table 2, Tables S1, S4. Color blocks without a white diagonal line represent gene fold change values (Table S1), color blocks containing a white diagonal line represent protein fold change values (Table 2, Table S4).

Figure S4 **Relative expression of enzymes determined as differentially expressed and associated with carbohydrate metabolism; the pentose phosphate and pentose glucuronate pathways in (A) the reference isolate ST24^WT^ and (B) the mutant isolate ST24^CHX^**.
