# Supplementary material for: Comparative analysis of Salmonella susceptibility and tolerance to the biocide chlorhexidine identifies a complex cellular defense network
Source: Front Microbiol. 2014 Aug 1;5:373. doi: 10.3389/fmicb.2014.00373 (PMC4117984; doi:10.3389/fmicb.2014.00373)
Supplement: Figure S1 — (A) Comparative respiration of ST24WT, and the chlorhexidine tolerant mutant, ST24CHX, on PM plates 1–10, containing energy sources and osmolytes. Substrates where a significant difference in the respiration between ST24WT and ST24CHX was recorded are indicated with arrows and details are provided in Table S6. (B) Comparative respiration of ST24WT and the chlorhexidine tolerant mutant, ST24CHX, on PM plates 11–20, containing antimicrobial compounds. Compounds where a significant difference in the respiration between ST24WT and ST24CHX were recorded, indicating a possible alteration in susceptibility, are indicated with arrows and details are provided in Table S6. [file DataSheet1.ZIP › Datasheet/Table S2.docx]

**Table S2:** Oligonucleotide primers used in this study.

| **Name** | **Sequence (5’-3’)** |
| --- | --- |
| *gyrA*_FP | CTT CGT AAT CTG TCA GCG GTT G |
| *gyrA*_RP | CGA TCA GTT CGG CGA TGA G |
| *rpoB*_FP | ACT GCT GCG TGC GAT CTT C |
| *rpoB*_RP | GCT GCA TCT CTT CGA TTT CCA G |
| *hilD*_FP | CAA CGA CTT GGC GCT CTC T |
| *hilD*_RP | TCT TCT GGC AGG AAA GTC AGG |
| *fadL*_FP | GTC AGA TTC CCA GCG ACA CC |
| *fadL*_RP | ACT TCC GAG CGG TAG GTC AG |
| *galP*_FP | GTG CTG GCA ACC TTC ATC G |
| *galP*_RP | CCA GAA TCC CCA TAC CAA TC |
| *flgG*_FP | ACC TGT CGC AGA CCA ACA ACA G |
| *flgG_*RP | GTT GAC CAT TCT GAT CCA CCT G |
| *folA*_FP | TGG ATC GCG TCA TCG GTA TG |
| *folA_RP* | CCA GGT GTG ACG TCC CAT G |
