# Supplementary material for: Comparative analysis of Salmonella susceptibility and tolerance to the biocide chlorhexidine identifies a complex cellular defense network
Source: Front Microbiol. 2014 Aug 1;5:373. doi: 10.3389/fmicb.2014.00373 (PMC4117984; doi:10.3389/fmicb.2014.00373)
Supplement: Figure S1 — (A) Comparative respiration of ST24WT, and the chlorhexidine tolerant mutant, ST24CHX, on PM plates 1–10, containing energy sources and osmolytes. Substrates where a significant difference in the respiration between ST24WT and ST24CHX was recorded are indicated with arrows and details are provided in Table S6. (B) Comparative respiration of ST24WT and the chlorhexidine tolerant mutant, ST24CHX, on PM plates 11–20, containing antimicrobial compounds. Compounds where a significant difference in the respiration between ST24WT and ST24CHX were recorded, indicating a possible alteration in susceptibility, are indicated with arrows and details are provided in Table S6. [file DataSheet1.ZIP › Datasheet/Table S3.docx]

**Table S3:** Summary of qRT-PCR and transcriptomic data for **(a)** the mutant isolate, ST24^CHX^, relative to the reference, ST24^WT^ and **(b)** the reference isolate ST24^WT^ chlorhexidine treated, relative to the same isolate without chlorhexidine.

(a)

| **Gene*** | **ST24^WT^** | | **ST24^CHX^** | | **Fold change** | | |
| --- | --- | --- | --- | --- | --- | --- | --- |
|  | Mean Ct value | Std. Dev^‡^ | Mean Ct value | Std. Dev^‡^ | *gyrA* normalised | *rpoB* normalised | Microarray^†^ |
| *gyrA* | 19.36 | 0.21 | 19.02 | 0.16 | - | - | - |
| *rpoB* | 18.95 | 0.1 | 18.39 | 0.23 | - | - | - |
| *hilD* | 19.1 | 1.17 | 21.60 | 2.73 | 0.16 | 0.17 | 0.18 |
| *fadL* | 21.33 | 0.19 | 28.39 | 1.43 | 0.01 | 0.02 | 0.18 |
| *galP* | 21.02 | 0.38 | 21.24 | 0.19 | 1.04 | 1.17 | 0.99 |
| *flgG* | 14.83 | 0.22 | 19.51 | 0.22 | 4.67 | 5.04 | 5.29 |
| *folA* | 21.64 | 0.25 | 20.77 | 0.27 | 1.12 | 1.25 | 2.27 |

*Details of all primers used are provided in Table S2.

^†^Microarray results are provided in Table S1.

^‡^Std Dev: standard deviation.

(b)

| **Gene*** | **ST24^WT^ un-treated** | | **ST24^WT^ chlorhexidine treated** | | **Fold change** | | |
| --- | --- | --- | --- | --- | --- | --- | --- |
|  | Mean Ct value | Std. Dev^‡^ | Mean Ct value | Std. Dev^‡^ | *gyrA* normalised | *rpoB* normalised | Microarray^†^ |
| *gyrA* | 19.36 | 0.21 | 19.72667 | 0.41 | - | - | - |
| *rpoB* | 18.95 | 0.1 | 18.17 | 0.31 | - | - | - |
| *hilD* | 19.1 | 1.17 | 20.51 | 0.04 | 0.44 | 0.48 | 0.37 |
| *fadL* | 21.33 | 0.19 | 20.35 | 0.19 | 0.90 | 0.82 | 0.6 |
| *galP* | 21.02 | 0.38 | 21.10 | 0.12 | 1.10 | 1.00 | 1.2 |
| *flgG* | 14.83 | 0.22 | 22.09 | 0.09 | 1.30 | 1.05 | 1.47 |
| *folA* | 21.64 | 0.25 | 21.28 | 0.24 | 1.50 | 1.33 | 1.67 |

*Details of all primers used are provided in Table S2.

^†^Microarray results are provided in Table S1.

^‡^Std Dev: standard deviation
