# Supplementary figures and images for: Comparative analysis of Salmonella susceptibility and tolerance to the biocide chlorhexidine identifies a complex cellular defense network
Source: Front Microbiol. 2014 Aug 1;5:373. doi: 10.3389/fmicb.2014.00373 (PMC4117984; doi:10.3389/fmicb.2014.00373)

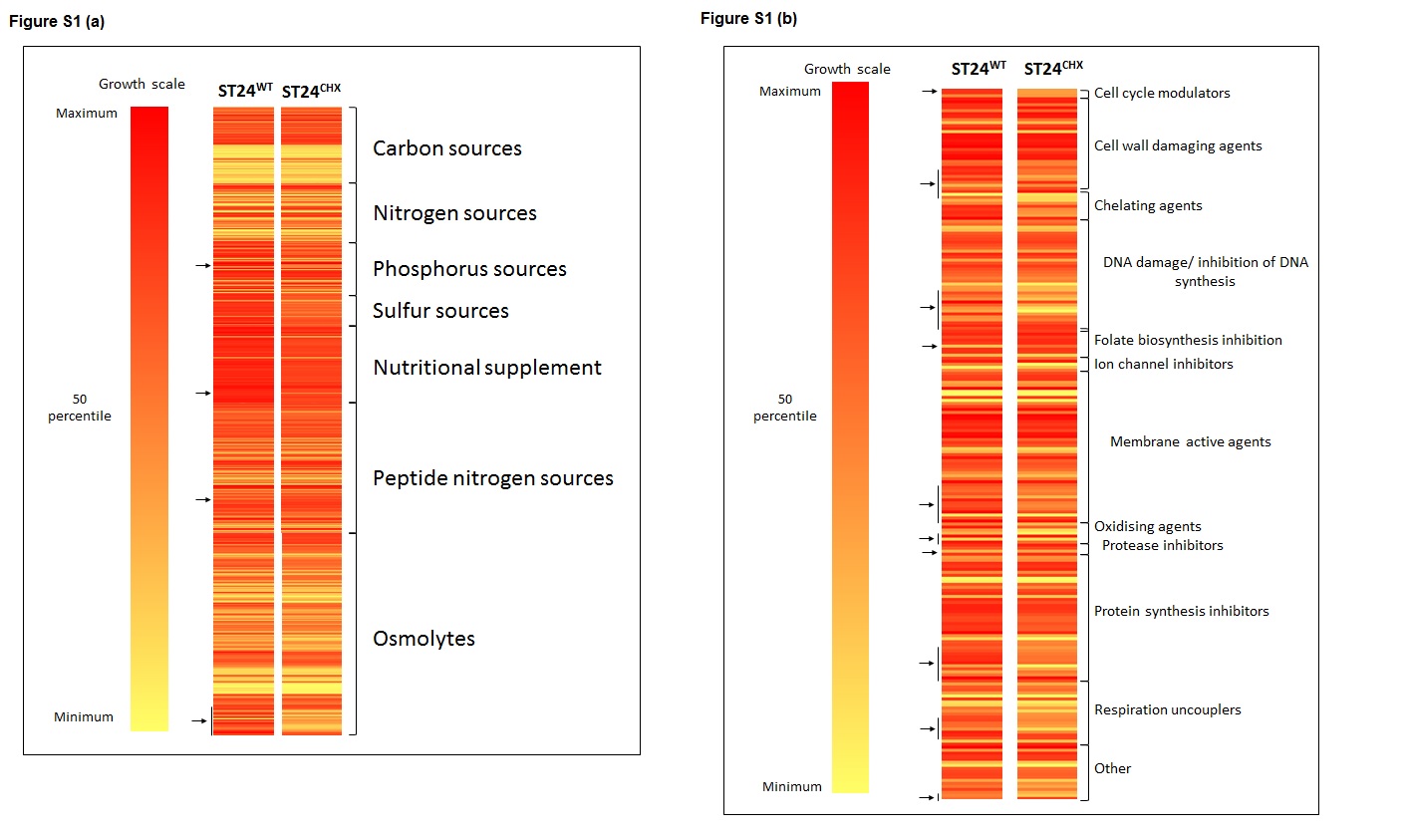

Supplement: Figure S1 — (A) Comparative respiration of ST24WT, and the chlorhexidine tolerant mutant, ST24CHX, on PM plates 1–10, containing energy sources and osmolytes. Substrates where a significant difference in the respiration between ST24WT and ST24CHX was recorded are indicated with arrows and details are provided in Table S6. (B) Comparative respiration of ST24WT and the chlorhexidine tolerant mutant, ST24CHX, on PM plates 11–20, containing antimicrobial compounds. Compounds where a significant difference in the respiration between ST24WT and ST24CHX were recorded, indicating a possible alteration in susceptibility, are indicated with arrows and details are provided in Table S6. [file DataSheet1.ZIP › Datasheet/Figure S1.JPEG]

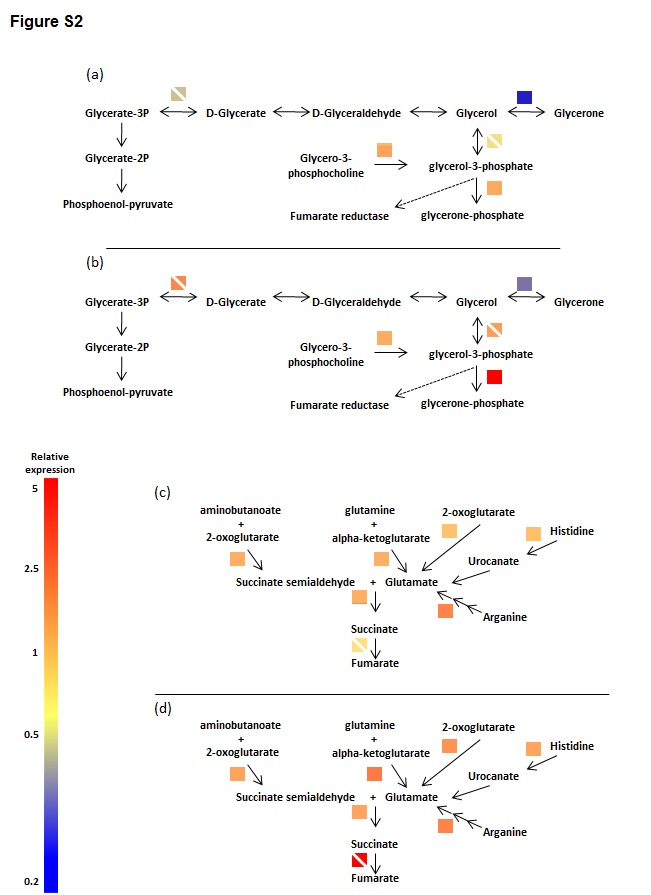

Supplement: Figure S1 — (A) Comparative respiration of ST24WT, and the chlorhexidine tolerant mutant, ST24CHX, on PM plates 1–10, containing energy sources and osmolytes. Substrates where a significant difference in the respiration between ST24WT and ST24CHX was recorded are indicated with arrows and details are provided in Table S6. (B) Comparative respiration of ST24WT and the chlorhexidine tolerant mutant, ST24CHX, on PM plates 11–20, containing antimicrobial compounds. Compounds where a significant difference in the respiration between ST24WT and ST24CHX were recorded, indicating a possible alteration in susceptibility, are indicated with arrows and details are provided in Table S6. [file DataSheet1.ZIP › Datasheet/Figure S2.JPEG]

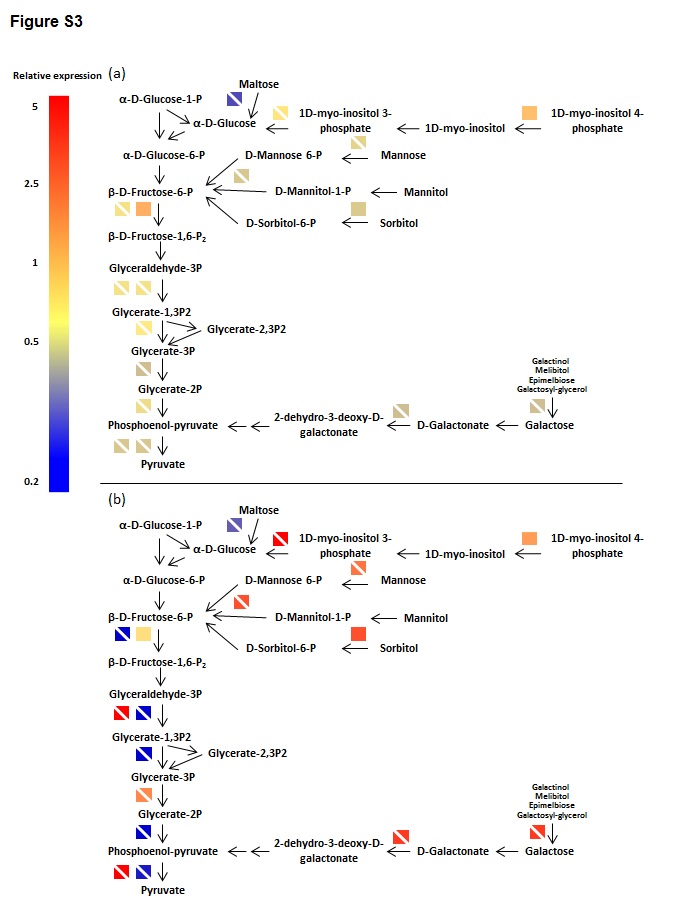

Supplement: Figure S1 — (A) Comparative respiration of ST24WT, and the chlorhexidine tolerant mutant, ST24CHX, on PM plates 1–10, containing energy sources and osmolytes. Substrates where a significant difference in the respiration between ST24WT and ST24CHX was recorded are indicated with arrows and details are provided in Table S6. (B) Comparative respiration of ST24WT and the chlorhexidine tolerant mutant, ST24CHX, on PM plates 11–20, containing antimicrobial compounds. Compounds where a significant difference in the respiration between ST24WT and ST24CHX were recorded, indicating a possible alteration in susceptibility, are indicated with arrows and details are provided in Table S6. [file DataSheet1.ZIP › Datasheet/Figure S3.JPEG]

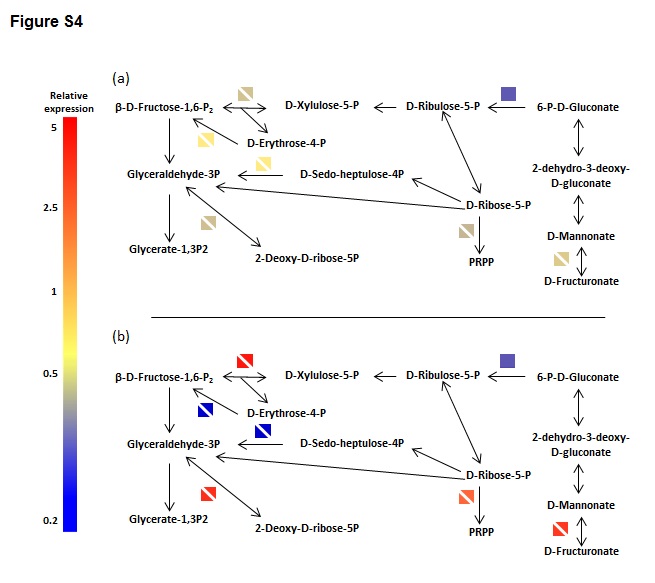

Supplement: Figure S1 — (A) Comparative respiration of ST24WT, and the chlorhexidine tolerant mutant, ST24CHX, on PM plates 1–10, containing energy sources and osmolytes. Substrates where a significant difference in the respiration between ST24WT and ST24CHX was recorded are indicated with arrows and details are provided in Table S6. (B) Comparative respiration of ST24WT and the chlorhexidine tolerant mutant, ST24CHX, on PM plates 11–20, containing antimicrobial compounds. Compounds where a significant difference in the respiration between ST24WT and ST24CHX were recorded, indicating a possible alteration in susceptibility, are indicated with arrows and details are provided in Table S6. [file DataSheet1.ZIP › Datasheet/Figure S4.JPEG]
